# A mathematical model to predict nanomedicine pharmacokinetics and tumor delivery

**DOI:** 10.1016/j.csbj.2020.02.014

**Published:** 2020-02-29

**Authors:** Prashant Dogra, Joseph D. Butner, Javier Ruiz Ramírez, Yao-li Chuang, Achraf Noureddine, C. Jeffrey Brinker, Vittorio Cristini, Zhihui Wang

**Affiliations:** a Mathematics in Medicine Program, Houston Methodist Research Institute, Houston, TX 77030, USA; b Department of Mathematics, California State University, Northridge, CA 91330, USA; c Department of Chemical and Biological Engineering, University of New Mexico, Albuquerque, NM 87106, USA; d UNM Comprehensive Cancer Center, University of New Mexico, Albuquerque, NM 87102, USA

**Keywords:** Cancer nanotherapy, Enhanced permeability and retention effect, Mechanistic mathematical modeling, PBPK, Pharmacokinetics, Sensitivity analysis

## Abstract

Towards clinical translation of cancer nanomedicine, it is important to systematically investigate the various parameters related to nanoparticle (NP) physicochemical properties, tumor characteristics, and inter-individual variability that affect the tumor delivery efficiency of therapeutic nanomaterials. Comprehensive investigation of these parameters using traditional experimental approaches is impractical due to the vast parameter space; mathematical models provide a more tractable approach to navigate through such a multidimensional space. To this end, we have developed a predictive mathematical model of whole-body NP pharmacokinetics and their tumor delivery *in vivo*, and have conducted local and global sensitivity analyses to identify the factors that result in low tumor delivery efficiency and high off-target accumulation of NPs. Our analyses reveal that NP degradation rate, tumor blood viscosity, NP size, tumor vascular fraction, and tumor vascular porosity are the key parameters in governing NP kinetics in the tumor interstitium. The impact of these parameters on tumor delivery efficiency of NPs is discussed, and optimal values for maximizing NP delivery are presented.

## Graphical abstract

**Figure:**
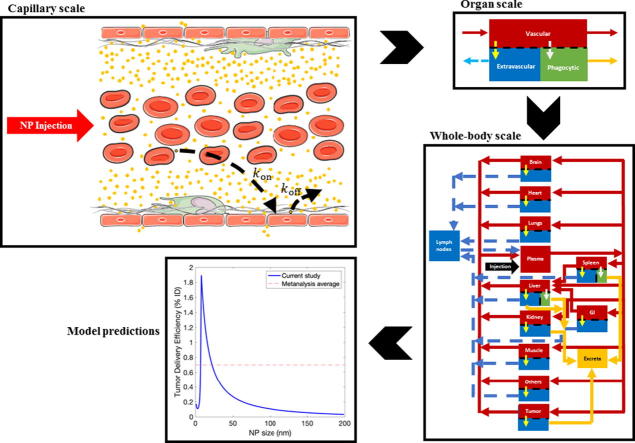

## 1 Introduction

Nanoparticle (NP)-mediated cancer therapy has been only moderately successful in clinical translation, and one of the main reasons for this lack of success is the low tumor delivery efficiency exhibited by NPs [1], [2], [3]. Despite their demonstrated potential to exploit the leaky tumor vasculature to passively accumulate in the tumor interstitium, a phenomenon described as the enhanced permeability and retention (EPR) effect [4], or their ability to actively target cancer cells following surface-functionalization [5], [6], [7], it has been found that across the board merely 0.7% ID (percent injected dose) of NPs accumulates in tumors [3]. Such low delivery efficiency can be primarily attributed to the clearance of NPs from the systemic circulation by mononuclear phagocytic system (MPS) organs, in particular the liver and spleen. Due to certain microanatomical and physiological features such as large vessel wall pores (also called sinusoidal fenestrations) and phagocytic cells (e.g. hepatic Kupffer cells and splenic macrophages) in the microvasculature of the liver and spleen, NPs are vulnerable to sequestration in these organs, thereby facing a major challenge following systemic injection [8], [9]. Nonetheless, these interactions depend upon the physicochemical properties of the NPs [10], [11], or the physiological state of the MPS [12], indicating that all NPs are not treated alike by the MPS, and manipulation of particle properties provides a way to regulate their sequestration by the MPS [13]. Similarly, clearance of NPs by the kidneys is NP size- and charge-dependent, and thus is another tunable mechanism of NP removal from the systemic circulation that can affect access of NPs to the tumor [14]. In a nutshell, the tumor delivery efficiency of NPs is not only dependent upon the properties of the tumor, but is also strongly dependent upon the properties of the NPs and the physiology of the MPS and kidneys.

To improve upon the status quo, the tumor delivery efficiency of NPs must be better understood in the context of both their systemic pharmacokinetics and the tumor microenvironment. While the divide and conquer strategy in the pre-clinical evaluation of this problem has improved our understaning of the importance of various physicochemical, biophysical, and physiological factors in isolation [10], [11], [12], [15], [16], [17], [18], the systemic-level phenomena can be drastically different when these isolated parts evolve together as a system; thus, a holistic investigation of this multidimensional parameter space is necessary to conclusively establish the importance of the key variables of interest. To this end, mathematical models can be a valuable tool to investigate *in silico* the role of NP-, tumor-, and individual-related parameters in determining the tumor deliverability of nanomaterials. While mathematical models developed in the past for investigation of *in vivo* NP dynamics have revealed valuable insights into NP-cellular interactions, effects of hemodynamics and hemorheology on intravascular NP transport, and the importance of NP properties and tumor microenvironment in delivery of NPs to the tumor [18], [19], [20], [21], [22], [23], [24], [25], [26], [27], [28], [81], they have either been limited in the scope of their spatiotemporal scale or the parameter space under investigation. Further, the physiologically based pharmacokinetic (PBPK) models developed so far, which are ideal for evaluating the disposition of NPs at the whole-body scale, have in our knowledge lacked an explicit tumor compartment, and instead have focused only on the whole-body biodistribution aspect of the problem [29], [30], [31], [32], [33], [34]. This limits the ability of these models to make predictions about the tumor delivery efficiency of the nanoformulations under investigation.

Thus, we have developed a tumor-compartment bearing PBPK model to investigate *in silico* the effects of NP properties, tumor variables, and individual physiological differences on the systemic bioavailability, MPS sequestration, tumor delivery, and excretion of NPs. Built on our extensive experience in mechanistic and PKPD modeling of nanomedicine and drug delivery in cancer [10], [16], [35], [36], [37], [38], this model is mechanistic in nature, making it capable of predicting systemic behaviors from properties of smaller parts, such that it can simulate the whole-body disposition and tumor delivery kinetics of NPs of any intended combination of physicochemical properties (including size, density, and degradation rate). The model has been validated with preclinical data, and local and global parameter sensitivity analyses have been conducted to rank the importance of various parameters pertinent to the problem of tumor delivery of NPs. We note that, while the therapeutic efficacy of NPs also depends upon their drug loading capacity (defined as the amount of drug that can be loaded into a given NP) and the IC_50_ of the payload, the investigation as is currently presented in this paper is only focused on the delivery efficiency of NPs.

## 2 Methods

### 2.1 Theoretical basis of the model

**Fig. 1:**
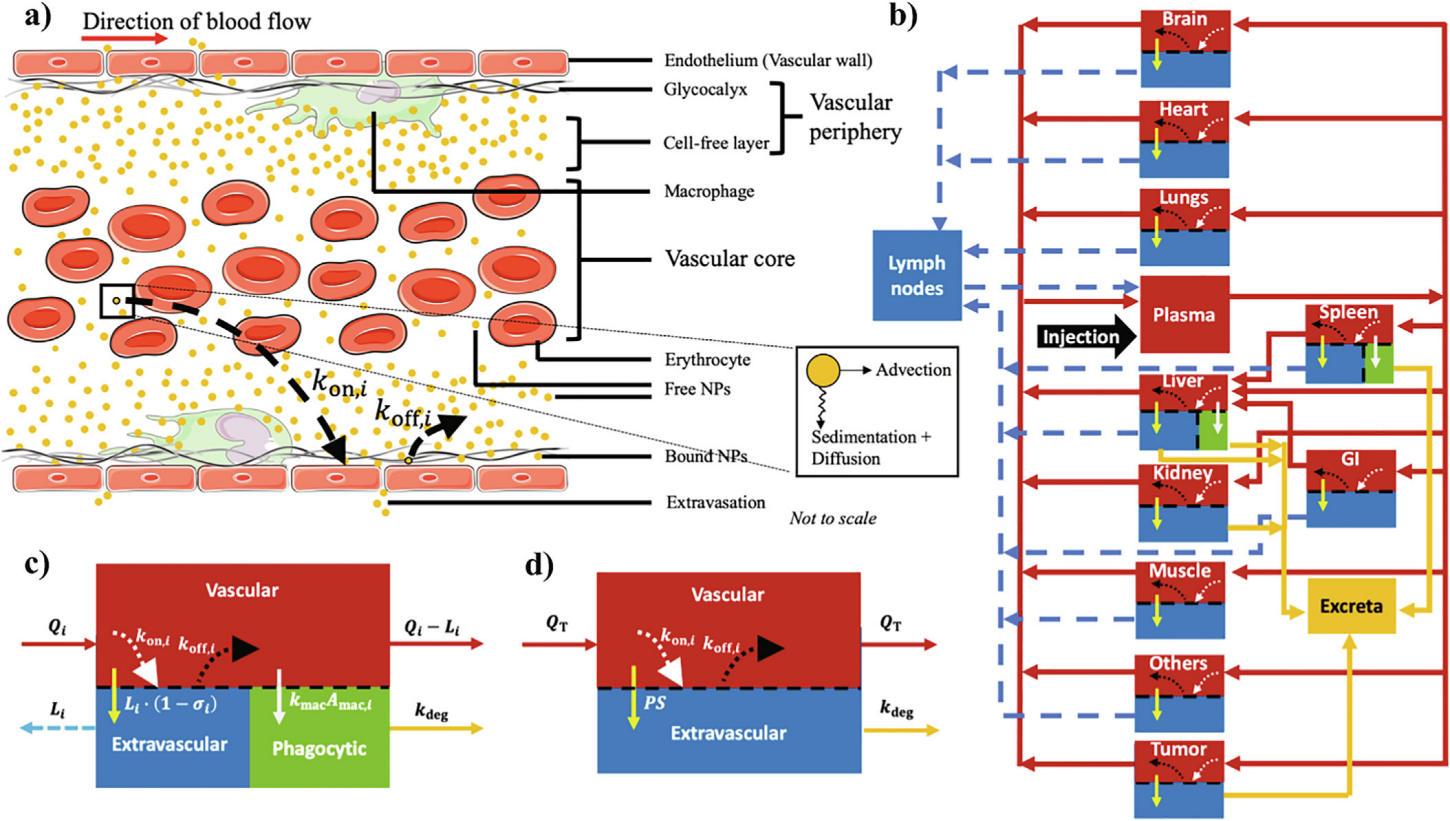
Mathematical model description. a) Dynamics of NPs at the capillary scale is shown such that they exhibit non-uniform radial distribution due to the effects of sedimentation, diffusion, and advection (inset). b) Structure of whole-body tumor-bearing PBPK model is shown. Notation- red arrows: plasma flow, dashed blue arrows: lymph flow, bright yellow arrows: extravasation, white arrows: phagocytosis, mustard arrows: excretion, dotted white arrows: NP deposition on the vascular wall ($k_{on,i}$), and dotted black arrows: NP dislodging from the vascular wall ($k_{off,i}$). GI denotes gastrointestinal tract. c) Schematic of a representative healthy compartment containing three sub-compartments: vascular, extravascular, and phagocytic. d) Schematic of tumor compartment containing vascular and extravascular sub-compartments. (For interpretation of the references to colour in this figure legend, the reader is referred to the web version of this article.)

Upon injection into the bloodstream, NPs are transported across the body via the vascular network through a highly dynamic microenvironment characterized by microscopic *nano-bio interactions* that govern the fate of NPs in terms of their global biodistribution kinetics and, more importantly, their delivery to the target site (in the current context, this is a solid vascular tumor) [20]. As shown in Fig. 1a, NP interactions with blood cells (erythrocytes in particular), with endothelium, or with endothelium-lining cells (macrophages) at the microvascular scale (capillary bed) play a critical role in NP pharmacokinetics, and are thus important components for effective mathematical modeling of NP transport kinetics through the vasculature of a given region of interest (ROI). Further, extravasation of NPs into the extravascular space (tissue interstitium) of the ROI, via either bulk transport or diffusion, can further modify the transport kinetics of NPs through the ROI. It is thus important to consider these interactions and transport processes in the development of a whole-body physiologically realistic model of NP pharmacokinetics and tumor delivery. To account for these interactions in our model, we characterize each process through mechanistic parameters derived through fundamental physical laws, as described below.

#### 2.1.1 NP deposition on the microvascular wall

As shown in Fig. 1a, NPs may not have a uniform radial distribution in the microvascular space; they may rather partition between the vascular core (cell-enriched layer) and the vascular periphery (cell-free layer and endothelial wall), with a preference for the periphery. We hypothesize that this partitioning is based on their tendency to undergo sedimentation, which maybe augmented by Brownian diffusion and shear-induced diffusion due to the presence of erythrocytes [24], [39], [40], [41], [42]. Once in the vascular periphery, NPs can either remain as free-flowing, or they may bind to the sticky glycocalyx covering the endothelial wall based on the interplay between their tendency to marginate from the cell-free layer towards the wall and their ability to dislodge from the endothelial glycocalyx, thereby governing the rate at which NPs pass through the capillary bed [13]. Thus, the intravascular partitioning of NPs and their tendency to remain as free-flowing or bound is affected by NP properties, hemorheology, and hemodynamic conditions in the microvasculature [22], [23], [25], which eventually affects the kinetics of NPs observed at the organ scale [43].

Assuming that NP motion from the core towards vessel the wall (radial direction) is due to gravitational and buoyant forces, we obtain the terminal sedimentation velocity v for a NP of radius r in the absence of longitudinal blood flow and erythrocytes via Stoke’s law:

(1) $$v=\frac{2}{9}\left(\frac{\rho_{np}-\rho_{p}}{\mu_{i}}\right)gr^{2}$$

where $\rho_{np}$ and $\rho_{p}$ are the density of NP and plasma, respectively; $\mu_{i}$ is the dynamic viscosity of blood in organ i, and g is the gravitational acceleration constant.

However, in the presence of blood flow and erythrocytes, the sedimentation velocity must be corrected for the effects of NP advection caused by blood flow (that will carry the particles along the length of the capillary), and with the velocity effects of diffusion. Heuristically, the stronger the advection of NPs, the faster will be their exit from the capillary bed (characteristic length equal to the length of a capillary l), and thus less time will be available to them to sediment and diffuse towards the endothelial wall [8]. However, with greater diffusivity, their approach will be faster towards the wall (characteristic length equal to the radius of a capillary R). Thus, correction to the sedimentation velocity is accomplished by dividing Eq. (1) by the Peclet number ($\frac{uR^{2}}{\mathrm{Dl}}$), where u is the average blood flow velocity in a capillary of characteristic length l (see section 4 of SI). Here, D denotes the effective diffusivity of NPs, obtained by summing diffusivity due to Brownian motion ($D_{B}$) and diffusivity due to shear-induced diffusion ($D_{S}$) caused by laminar flow in a cylindrical vessel under no-slip boundary conditions and interaction with erythrocytes. We used the Stokes-Einstein equation to calculate $D_{B}$, and an analytical expression developed by Xu et al. [40] to calculate $D_{S}$ as:

(2) $$D_{S}=0.3\beta^{2}\dot{\gamma }H^{2}$$

where β is the radius of erythrocytes, $\dot{\gamma }$ is the shear rate (defined in section 4 of SI), and H is the hematocrit: the volume fraction of red blood cells in the blood.

Further, by dividing the corrected sedimentation velocity of NPs by the characteristic length of sedimentation (radius of microvessel, R) we obtain the $k_{on,i}$ parameter (time^−1^) that characterizes the rate of deposition of NPs on the microvascular wall:

(3) $$k_{on,i}=\frac{2}{9}\left(\frac{\rho_{np}-\rho_{p}}{\mu_{i}}\right)\frac{gr^{2}Dl}{uR^{3}}$$

The relative magnitude of sedimentation, Brownian diffusion, erythrocyte-enhanced diffusion, and advection depend upon NP properties, blood characteristics, and flow conditions, and the dominant process(es) may change based on the size of therapeutic NPs delivered. In order to obtain a generalized model of NP pharmacokinetics that is applicable across a wide range of NP sizes (nm-μm) and physiological conditions, we included all of these processes in our model.

#### 2.1.2 NP dislodging from the microvascular wall

NPs that have marginated towards the microvascular wall are either endocytosed by endothelium-lining macrophages, as discussed next, or remain non-specifically bound on the endothelial wall. However, the latter can be overcome, and the particles can reenter the vessel lumen to continue their longitudinal transport if the NPs can diffuse back through the thickness of the sticky glycocalyx covering the endothelial wall (characteristic diffusion length equal to the thickness of glycocalyx $l_{g}$). This provides us with a measure of the rate of dislodging $k_{off,i}$ (time^−1^) from the endothelium as:

(4) $$k_{off,i}=D_{B}/l_{g}^{2}$$

Although NP dislodgement from the endothelial wall maybe affected by the strength of specific interactions between NP-surface ligands and endothelial cell surface receptors [44], we assume for simplicity that the relative tendency of NPs to approach the endothelial wall and diffuse away from the wall are respectively governed by $k_{on,i}$ and $k_{off,i}$. We note that inclusion of the strength of microscopic NP-cellular interactions is currently out of the scope of this model.

#### 2.1.3 NP phagocytosis

To quantify the rate constant $k_{mac}$ (time^−1^) of NP phagocytosis by endothelium-lining macrophages, we take the inverse of the NP wrapping time of macrophages, estimated by equating the mechanical work performed by motor proteins for wrapping NPs against the elastic energy of the macrophage membrane. We implement this using the following expression derived by Lunov et al. [45]:

(5) $$k_{mac}=p/4\pi r^{2}\gamma$$

where p is the power of motor proteins and γ is the surface tension of the membrane.

#### 2.1.4 NP extravasation via bulk transport or diffusion

NPs in the vasculature of a healthy or tumorous tissue can extravasate across the vascular endothelium via pores or fenestrations in the vascular wall to enter the tissue interstitium (extravascular space). Every healthy tissue has a characteristic pore size (radius, $r_{pore,i}$) associated with its vascular wall that controls the passage of fluids and solutes [46]. NPs can cross the endothelial wall through *bulk transport* with the effluxing lymph fluid, as regulated by the size-dependent impedance to particle extravasation through fenestrations, which is characterized by the reflection coefficient $\sigma_{i}$. The reflection coefficient for a NP is given by the following empirical relation [47]:

(6) $$\sigma_{i}=1-\left\{1-{\left[1{-\left(1-\alpha_{i}\right)}^{2}\right]}^{2}\right\}G+\frac{16}{9}\alpha_{i}^{2}{\left(1-\alpha_{i}\right)}^{2}F$$

where $\alpha_{i}$ is the ratio of NP size to pore size, i.e. $r/r_{pore,i}$; G and F are decreasing hydrodynamic functions of $\alpha_{i}$ (given in section 2 of SI (Eqs. (S36), (S37))). A detailed analysis of the vascular reflection coefficient and vasculature permeability (Eq. (7), below) as a function of NP size is presented in Fig. S4.

Tumors have much larger pores in their vascular walls relative to healthy tissue, that are a result of poorly developed neo-angiogenic blood vessels [4], [48]. Although this makes the tumor vessels highly permeable, it also causes excessive leakage of lymph into the tumor interstitium [49], [50], [51]. Further, due to solid stress generated in a growing tumor, lymph vessles tend to be non-functional due to physical compression, thereby leading to poor lymphatic drainage [52]. Both of these factors lead to elevated tumor interstitial fluid pressure that reduces the hydrostatic pressure difference between vasculature and interstitium; thus bulk transport of NPs is hampered, and the only feasible means of extravasation of NPs in tumors is *diffusion* [53], [54]. Diffusion-dependent extravasation of NPs across the leaky tumor vascular wall is characterized by the permeability (P)- surface area (S) product (P∙S, dimensions: volume·time^−1^), and we use it to model the passive targeting of NPs to solid tumors (EPR effect). The expression for permeability as obtained from Mescam et al. [47] is:

(7) $$P=\phi ∙\left(1-\alpha_{T}\right)FD_{B}/l_{w}$$

where ϕ denotes the porosity of the tumor vessels and is defined as the area of pores in a unit area of the microvessels, and $l_{w}$ represents the thickness of the vessel wall. The calculation of total tumor microvascular surface area S is given in Section 3 of SI.

### 2.2 PBPK model development

To predict the global disposition kinetics of NPs, we used a PBPK modeling framework and incorporated the previously described microscopic mechanisms of NP interactions in the organ microvasculature [55]. The model is composed of the following compartments, representing the major organs or tissues of interest: brain, heart, lungs, plasma, liver, spleen, gastrointestinal tract, kidneys, muscle, others, lymph nodes, and a facultative tumor (see Fig. 1b). The “others” represents a lumped compartment comprising bones, glands, and adipose tissue. Each healthy compartment is divided into a vascular and an extravascular sub-compartment, with the MPS organ compartments (liver and spleen) containing an additional macrophage sub-compartment. The model is formulated as a system of ordinary differential equations (ODEs) that are based on conservation of mass and the law of mass action. Here, we detail the equations that describe the NP distribution kinetics in a *representative* healthy organ compartment (containing all the three sub-compartments; Fig. 1c) and in the tumor compartment (Fig. 1d). For the complete system of equations, refer to section 1 of SI (Eqs. (S1)–(S35)).

#### 2.2.1 Healthy organ compartment

##### 2.2.1.1 Vascular sub-compartment

At the organ scale, as shown in Fig. 1c, the vascular sub-compartment, which represents the intravascular volume of the tissue, is fed with NPs via the incoming blood at plasma flow rate $Q_{i}$. Once in the vascular space, particles can either efflux into the interstitial space via bulk transport with the flowing lymph at the lymph flow rate $L_{i}$, or can exit the vascular space to rejoin the systemic circulation at a rate $Q_{i}-L_{i}$. These three transport processes are typically used to model the kinetics of NPs or antibodies in the vascular sub-compartment. However, as discussed previously, the non-homogenous distribution of NPs within the vascular space due to sedimentation and their interaction with erythrocytes, macrophages, and the endothelial wall can affect the kinetics of NPs passing through the microvasculature of a given organ.

As an improvement over conventional PBPK models, to capture the vascular dynamics of NPs more appropriately, we introduced the mechanistic $k_{on,i}$ (Section 2.1.1) and $k_{off,i}$ (Section 2.1.2) parameters in our model to incorporate the physical phenomena of NP deposition on the vessel wall and dislodging from the vessel wall, respectively. Further, since the particles in the vascular periphery may be captured by macrophages in the MPS organs, we characterize this process by the mechanistic parameter $k_{mac}$ (Section 2.1.3). Thus, by classifying NPs in the vascular sub-compartment into *free* and *bound* particles, we obtain the following ODEs for NP mass kinetics in the vascular space:

Free NPs

(8) $$\begin{array}{c} V_{v,i}\frac{dC_{v,i}^{f}}{\mathrm{dt}}={Q_{i}C}_{P}-\left(Q_{i}-L_{i}\right)C_{v,i}^{f}-{L_{i}∙\left(1-\sigma_{i}\right)C}_{v,i}^{f}-{k_{on,i}V_{v,i}C}_{v,i}^{f}+{k_{off,i}V_{v,i}C}_{v,i}^{b},\\ {V_{v,i}C}_{v,i}^{f}\left(0\right)=0 \end{array}$$

Bound NPs

(9) $$V_{v,i}\frac{dC_{v,i}^{b}}{\mathrm{dt}}={k_{on,i}V_{v,i}C}_{v,i}^{f}-{k_{off,i}V_{v,i}C}_{v,i}^{b}-{k_{mac}A_{mac,i}V_{v,i}C}_{v,i}^{b},\;{V_{v,i}C}_{v,i}^{b}\left(0\right)=0$$

where $C_{v,i}^{f}$ and $C_{v,i}^{b}$ represent the concentration of free-flowing and bound NPs in the vasculature of organ i, respectively; $C_{P}$ is the NP concentration in the plasma compartment; $V_{v,i}$ is the vascular volume of organ i; and $A_{mac,i}$ represents the area fraction of macrophages in the microvasculature of organ i, discussed below.

##### 2.2.1.2 Extravascular sub-compartment

As discussed before, the extravascular sub-compartment, representing the extravascular volume of the organ, receives NPs from the vascular sub-compartment through bulk transport of NPs along with the lymph fluid movement, and regulated by the NP to vascular pore size-dependent reflection coefficient. Thus, we have:

(10) $$V_{e,i}\frac{dC_{e,i}}{\mathrm{dt}}={L_{i}∙\left(1-\sigma_{i}\right)C}_{v,i}^{f}-{L_{i}C}_{e,i},\;{V_{e,i}C}_{e,i}\left(0\right)=0$$

where $C_{e,i}$ is the concentration of NPs in the extravascular sub-compartment of volume $V_{e,i}$.

##### 2.2.1.3 Phagocytic sub-compartment

NPs that are interacting with the vascular wall are prone to phagocytosis by macrophages lining the vascular wall in the MPS. The rate of uptake of NPs by the macrophages can be represented as: $\frac{dN_{p,i}}{\mathrm{dt}}={k_{mac}V_{v,i}C}_{v,i}^{b}$, where $N_{p,i}$ is the mass of phagocytized NPs. However, in reality not all of these NPs may be accessible by macrophages for engulfment due to lack of proximity, and in order to correct for this we multiply $C_{v,i}^{b}$ by the dimensionless term $A_{mac,i}$ (area fraction of macrophages in the microvasculature of organ i). Thus, $A_{mac,i}C_{v,i}^{b}$ represents the concentration of vascular bound NPs that are in close proximity of macrophages and are available to undergo phagocytosis. Thus, rate of NP phagocytosis can now be represented as:

(11) $$\frac{dN_{p,i}}{\mathrm{dt}}={k_{mac}A_{mac,i}V_{v,i}C}_{v,i}^{b}$$

We further assume that phagocytosed NPs are degraded (and excreted) at a rate characterized by a first order rate constant $k_{deg}$. Thus, the kinetics of the phagocytic sub-compartment can be represented as:

(12) $$\frac{dN_{p,i}}{\mathrm{dt}}={k_{mac}A_{mac,i}V_{v,i}C}_{v,i}^{b}-{k_{deg}N}_{p,i}\;\;N_{p,i}\left(0\right)=0$$

Note that organs other than the MPS organs do not have a phagocytic sub-compartment due to the anatomical localization of macrophages in the extravascular space of such organs, so these macrophages do not have direct access to NPs in the vasculature, i.e. $A_{mac,i}\approx 0$. Refer to SI for the detailed equations of all the organs (Eqs. (S1)–(S35)).

Additionally, excretion of NPs from the extravascular sub-compartments in the kidneys and liver is governed by glomerular filtration rate (GFR), urine (U), and bile (B) flow rates, which are described in greater detail in the SI.

#### 2.2.2 Tumor compartment

As shown in Fig. 1d, while the tumor compartment follows the same general structure as a healthy compartment without the phagocytic sub-compartment, the bulk transport-mediated extravasation of NPs from the vascular to the extravascular sub-compartment is replaced by diffusion-dependent transport. Also, we assume that NPs sequestered in the tumor interstitium undergo degradation (and excretion) at the same rate $k_{deg}$ as in the MPS macrophages. Thus, the mass kinetics of NPs in the vascular and extravascular sub-compartments of the tumor can be represented as:

##### 2.2.2.1 Vascular sub-compartment

Free NPs

(13) $$V_{v,T}\frac{dC_{v,T}^{f}}{\mathrm{dt}}={Q_{T}∙(C}_{P}-C_{v,T}^{f})-{P∙S∙C}_{v,T}^{f}-{k_{on,T}V_{v,T}C}_{v,T}^{f}+{k_{off,T}V_{v,T}C}_{v,T}^{b}\;{V_{v,T}C}_{v,T}^{f}\left(0\right)=0$$

Bound NPs

(14) $$V_{v,T}\frac{dC_{v,T}^{b}}{\mathrm{dt}}={k_{on,T}V_{v,T}C}_{v,T}^{f}-{k_{off,T}V_{v,T}C}_{v,T}^{b}\;{V_{v,T}C}_{v,T}^{b}\left(0\right)=0$$

##### 2.2.2.2 Extravascular (tumor interstitum) sub-compartment

(15) $$\frac{dN_{e,T}}{\mathrm{dt}}=P∙S∙C_{v,T}^{f}-k_{deg}N_{e,T},\;N_{e,T}\left(0\right)=0$$

where $N_{e,T}$ is the mass of NPs in the tumor interstitium.

### 2.3 Model parameterization and validation

Several physiological parameters were known a priori from the literature (Table 1, Table 3), and the rest were derived through mathematical models discussed above (Table 2). Equations used for estimation of these parameters are given in SI.

**Table 1:** List of model parameters and physical constants known a priori. Notation: plasma (i=P), liver (i=L), kidneys (i=K), lungs (i=LU), spleen (i=S), brain (i=B), heart (i=H), gastro intestinal tract (i=GI), muscle (i=M), others (i=O).

Parameter	Description (Units)	Value (References)									
		Organ i									
		P	L	K	LU	S	B	H	GI	M	O
$f_{WT,i}$	Fractional body weight of organ i	0.0589 [64]	0.05 [64]	0.015 [64]	0.01 [64]	0.01 [64]	0.03 [64]	0.01 [64]	0.04 [64]	0.3 [64]	0.03 [64]

$f_{v,i}$	Vascular fraction of organ i (for calculation of vascular volume of organ i, Eq. (S44))	–	0.21 [64]	0.16 [64]	0.36 [64]	0.22 [64]	0.04 [64]	0.26 [64]	0.2 [64]	0.04 [64]	0.01 [64]
$f_{CO,i}$	Fractional cardiac output to organ i (for calulation of plasma flow rate $Q_{i}$ to organ i, Eq. (S43))	–	0.021* [64]	0.141 [64]	1 [64]	0.02 [64]	0.02 [64]	0.051 [64]	0.133 [64]	0.278 [64]	0.01 [64]
$r_{pore,i}$	Radius of fenestrations in microvessel wall (nm)	–	140 [46]	4 [46]	2.5 [46]	2500 [46]	0.5 [46]	2.5 [46]	2.5 [46]	2.5 [46]	2.5 [46]
CO	Cardiac output ($ml∙h^{-1}$)	4217 (computed from Eq. (S42) for reference rat of weight 200 g) [64]									
$L_{i}$	Lymph flow rate of organ i ($ml∙h^{-1}$)	Lymph flow rates are 1/500 times of plasma flow rates [65], [80].									
GFR	Glomerular filtration rate ($ml∙h^{-1}$)	78.6 [66]									
U	Urine flow rate ($ml∙h^{-1}$)	2.0833 [66]									
B	Bile flow rate ($ml∙h^{-1}$)	0.9375 [66]									
$l_{w}$	Thickness of tumor microvessel wall (μm)	5 [26]									
l	Length of capillary (μm)	1000 (assumed)									
R	Radius of capillary (μm)	5 [67]									
$r_{tumor}$	Radius of tumor (mm)	5 (assumed)									
$l_{g}$	Thickness of glycocalyx (nm)	100 [68]									
$\mu_{i}$	Dynamic viscosity of blood in healthy organs (cP)	4 [69], [70]									
$f_{cap}$	Capillary volume fraction	0.55 [71]									
β	Radius of erythrocyte (μm)	3.6 [40]									
$r_{mac}$	Radius of macrophage (μm)	15 [72]									
p	Power of cellular motor proteins (W)	10^−17^[45]									
γ	Surface tension of cellular membrane (m$N\cdot m^{-1}$)	0.06 [45]									
$k_{B}$	Boltzmann constant ($JK^{-1}$)	1.38*10^−23^									
T	Body temperature (K)	310									
g	Gravitational acceleration constant (m⋅s^−2^)	9.8									
$\rho_{p}$	Plasma density (g⋅cm^−3^)	1									

*Contribution from hepatic artery.

**Table 2:** List of estimated model parameters.

Parameter	Description (Units)	Calculation	Value			
			Tumor		Healthy	
			Estimated	Literature (Reference)	Estimated	Literature (Reference)
u	Capillary blood flow velocity (μm⋅s^−1^)	Eq. (S40)	30	0–200 [73]	553	100–1000 [8], [70], [74]
$Q_{cap,i}$	Capillary blood volumetric flow rate (nl⋅min^−1^)	Eq. (S39)	0.15	0.21 (ischemia) [75]	2.58	2.4–3.47 [8], [70]
$\dot{\gamma }$	Capillary shear rate (s^−1^)	Eq. (S41)	16	0–30 [73]	116	100–1000 [61], [70], [74]
$\sigma_{i}$	Reflection coefficient	Eqs. (6), (S36), (S37)	n/a		Liver: 0.43, Spleen: 0.0025, Others: 1 (reference NP size 10 nm); also see Fig. S4a	Liver, spleen: 0.85 Others: 0.9–0.99 (reference NP size ~ 10 nm) [65]
P	Permeability (mm⋅s^−1^)	Eq. (7)	1.1 × 10^−7^ (reference NP size 90 nm); 1.27 × 10^−6^ (reference NP size 9.4 nm); also see Fig. S4b	2 × 10^−7^ (reference NP size 90 nm) [76]; 2.82 × 10^−6^ (reference NP size 9.4 nm) [77]	n/a	
S	Surface area (mm^2^/mm^3^)	Section 3 of SI	40	34–45 [78]	n/a

**Table 3:** List of parameters for sensitivity analysis. Perturbed parameter values were chosen randomly from a uniform distribution within the ±99% range around their respective literature-based mean values.

Parameter	Description (Units)	Value (References)
*NP-related parameters*		
r	Nanoparticle radius (nm)	50
$\rho_{np}$	Nanoparticle density (g⋅cm^−3^)	2 [39]
$k_{deg}$	Nanoparticle degradation rate (h^−1^)	0.01 [16]
*Tumor-related parameters*		
$Q_{T}$	Tumor blood flow rate (ml⋅g^−1^⋅min^−1^)	0.1 [79]
$\mu_{T}$	Tumor blood viscosity (cP)	7.42 [61]
$f_{v,T}$	Tumor vascular fraction†	0.1 [61]
$r_{pore,T}$	Tumor vascular pore radius (nm)	850 [48]
ϕ	Tumor vascular porosity	0.001 [48]
*Individual-related parameters*		
$A_{mac,L}$	Area fraction of liver vasculature occupied by Kupffer cells (section 7 of SI)	0.5 [29]
$A_{mac,S}$	Area fraction of spleen vasculature occupied by splenic macrophages (section 7 of SI)	0.1 [29]
H	Hematocrit (%)	45 [61]

† Volume fraction of tumor occupied by vasculature.

The PBPK model (without the tumor compartment) was first validated with longitudinal *in vivo* data extracted from our previously published study on NP pharmacokinetics [10]. In this study, highly stable, monodisperse, radiolabeled mesoporous silica NPs were administered intravenously into rats, following which the animals were imaged over time until 24 h post injection. The images were quantified to estimate the radioactivity concentration (defined as percent of injected dose per gram, %ID^.^g^−1^) in various organs of the body as a surrogate for NP concentration kinetics. We used the data from this study for neutrally charged NPs ranging in hydrodynamic size from ~45 nm to ~160 nm to quantitatively compare the model simulation predictions with the observed data. The quality of predictions was assessed by conducting a correlation analysis between model predictions and the observed data. All the analyses were performed in MATLAB R2018a.

### 2.4 Model analysis

To investigate the significance of how various model parameters affect NP pharmacokinetics and their delivery to the tumor, we conducted both local (LSA) and global (GSA) sensitivity analyses. The effects of parameters were quantified based on area under the curve (${AUC}_{0-\infty }$) of plasma, MPS, tumor interstitum, and excretion curves.

#### 2.4.1 Local sensitivity analysis (LSA)

LSA was conducted by perturbing one parameter at a time over a range ±99% of its reference value (these are shown in Table 3), while the remaining parameters were held constant at their respective reference values. Each parameter was tested at 1000 levels within the above range, and four model outputs of interest were examined: ${AUC}_{0-\infty }^{plasma}$, ${AUC}_{0-\infty }^{MPS}$, ${AUC}_{0-\infty }^{tumor}$, and ${AUC}_{0-\infty }^{excreta}$. The effect of each parameter perturbation was quantified by measuring the sensitivity coefficient (SC) as:

(16) $$SC=\frac{\left(AUC_{0-\infty }^{\text{'}}-AUC_{0-\infty }\right)/AUC_{0-\infty }}{({\mathrm{Par}}^{\text{'}}-\mathrm{Par})/\mathrm{Par}}$$

where ${AUC}_{0-\infty }$ and ${AUC}_{0-\infty }^{\text{'}}$ represent the AUC of a given compartment before and after perturbation, respectively; Par refers to the reference value of the parameter under investigation, and ${\mathrm{Par}}^{\text{'}}$ represents its perturbed value. Trapezoidal numerical integration via built-in MATLAB function *trapz* was used to estimate the AUCs. A larger value of the SC denotes greater significance of the parameter for the given model output. The parameters were then ranked for each model output based on the maxima of the absolute value of SCs obtained for parameter perturbations over the ±99% range.

#### 2.4.2 Global sensitivity analysis (GSA)

GSA involved simultaneous perturbation of all eleven tested parameters within their respective ±99% range around the reference value (Table 3). We followed a sampling-based GSA workflow described by Wang et al. [56], [57] to conduct the analysis. Briefly, Latin hypercube sampling (LHS) was used to obtain a sample of 5000 sets of parameters from the multidimensional parameter space under investigation. Ten such samples were generated, unlike the bootstrapping done by Wang et al. A simulation was run for each set of parameters to estimate the four model outputs (${AUC}_{0-\infty }^{plasma}$, ${AUC}_{0-\infty }^{MPS}$, ${AUC}_{0-\infty }^{tumor}$, and ${AUC}_{0-\infty }^{excreta}$). On each sample, multivariate linear regression analysis (MLRA), partial rank correlation analysis (PRCA), and analysis of variance (ANOVA) was conducted to obtain the regression coefficient from MLRA, partial correlation coefficient from PRCA, and F-value from ANOVA as a measure of the sensitivity index (SI) for each model output. Ranking of parameters was then obtained via one-way ANOVA and Tukey’s test on the SIs for ten samples for each technique. Finally, a weighted parameter ranking from the three techniques was obtained as outlined in Wang et al. [56]. All analyses were performed in MATLAB R2018a.

## 3 Results and discussion

### 3.1 Model is independently validated with a NP biodistribution dataset

In our model, the parameters were either known a priori or estimated through mathematical models (see Sections 2.1–2.3). We thus directly move to the model validation step without performing any regression-based model calibration procedures. The PBPK model (without the tumor compartment) was used to simulate the whole-body disposition kinetics of NPs of different sizes (46 nm, 69 nm, 113 nm, and 162 nm) following intravenous administration into the plasma compartment of rats. The selected sizes match the hydrodynamic sizes of NPs for which *in vivo* data used for model validation was available in the literature [10]. Details of model parameters used in these simulations are given in Table 1, Table 2. The model system of ODEs was solved numerically in MATLAB as an initial value problem, using the built-in stiff ODE solver *ode15s*, with the plasma compartment containing 100% ID at time t = 0 and the remaining compartments containing 0% ID at the initial time.

**Fig. 2:**
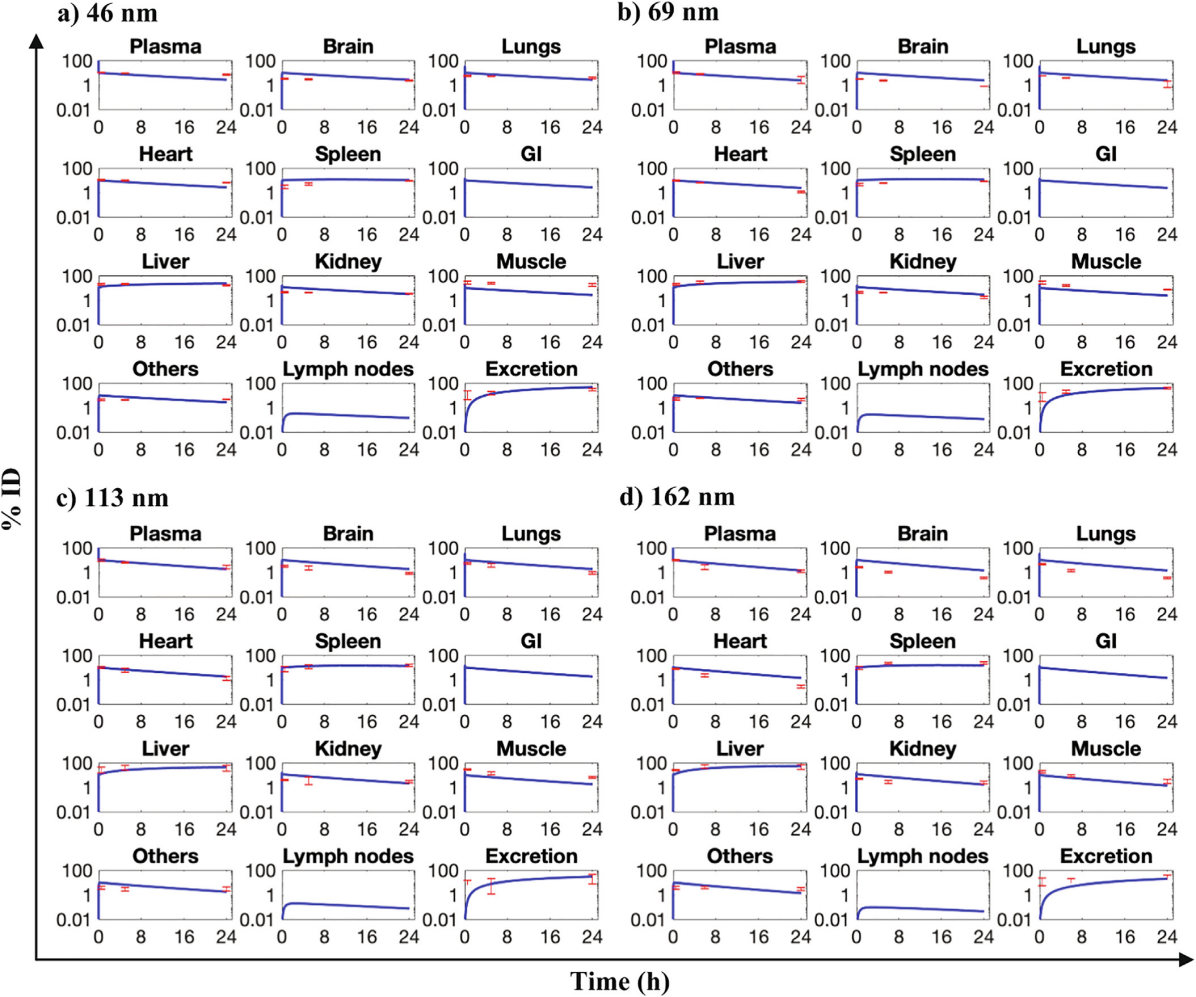
*In vivo* model validation. Model simulation outputs for NPs of size a) 46 nm, b) 69 nm, c) 113 nm, and d) 162 nm are shown as blue lines in various model compartments. Red dots and error bars represent mean ± S.D. of the experimental data from the literature for mesoporous silica NPs of matching hydrodynamic sizes (n = 4 animals per group). Note: y-axes are in logarithmic scale. (For interpretation of the references to colour in this figure legend, the reader is referred to the web version of this article.)

As shown in Fig. 2, the model solution correctly represents the initial conditions, and as calculated, the sum of NP mass (% ID) across all compartments in a given simulation at any time point is 100% ID, i.e., conservation of mass is upheld in the model as was intended. Importantly, the model predictions of NP mass kinetics over 24 h are in close agreement with the experimental observations in the majority of compartments for all four NP sizes (Fig. 2, red points), as confirmed through a Pearson correlation coefficient *R* > 0.94 (Fig. S1), thereby indicating good predictive performance of the model. Because *in vivo* data was not available for all modeled compartments, model outputs of gut and lymph node compartments could not be evaluated for their accuracy.

Further, becuase the tumor compartment was not included in the above validation, we indirectly validated the physiological consistency of the tumor compartment by comparing the estimated parameters pertinent to the tumor compartment with physiological values available in the literature. As seen in Table 2, all estimated model parameters, including the ones for healthy compartments, are within their physiologically viable range, thereby lending credibility to the simulation results of the tumor-bearing model (discussed next).

### 3.2 Parameter analysis

**Fig. 3:**
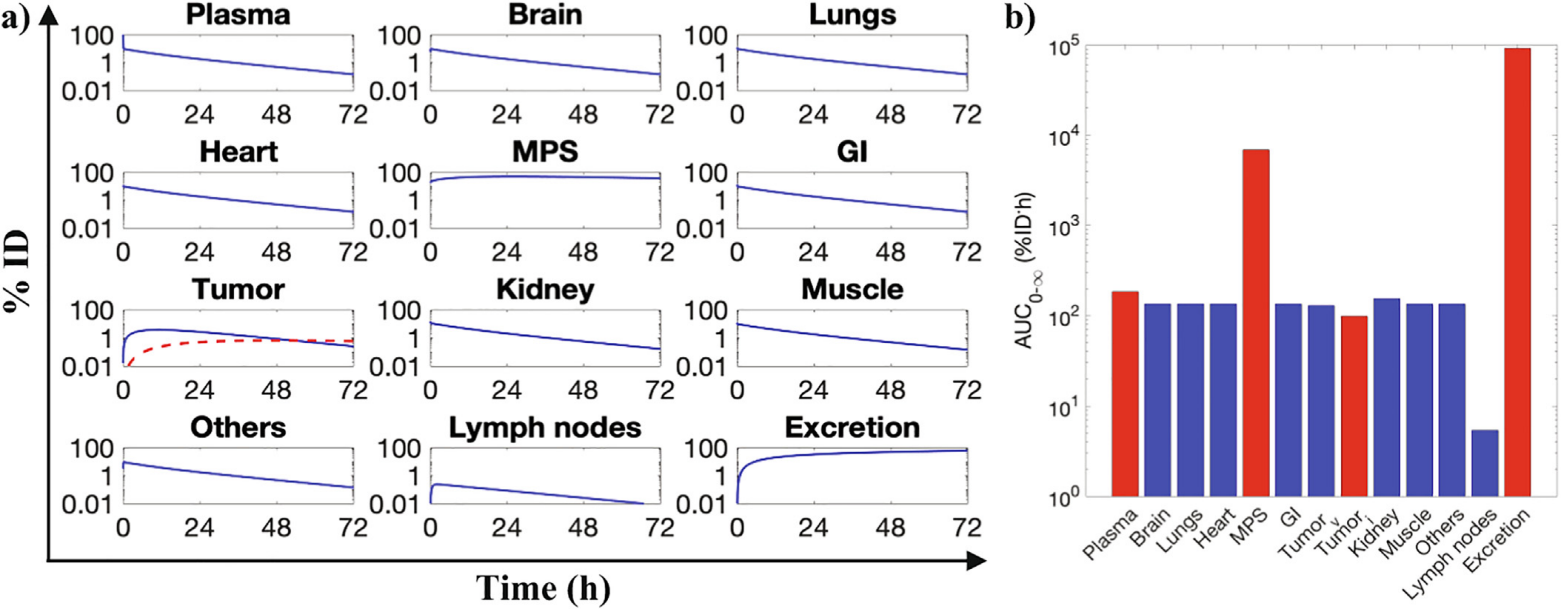
Reference behavior of tumor-bearing model. a) Model simulation for a NP of size 100 nm, as a reference for the sensitivity analysis, is shown. Dashed red line and solid blue line in the tumor panel represent NP mass kinetics in the tumor interstitium and tumor vascular space, respectively. Solid blue lines in the remaining panels denote the total NP mass kinetics in the given compartment. All reference values are shown in Table 1, Table 3; tumor was 1 cm in diameter in the simulation run shown. b) Area under the curves for the model compartments from 0 to ∞ (1000 h) are shown. Bars corresponding to compartments used in the sensitivity analyses are colored in red. Note: Tumor_v_ and Tumor_i_ refer to tumor vasculature and tumor interstitium, respectively; MPS denotes liver and spleen compartments combined. (For interpretation of the references to colour in this figure legend, the reader is referred to the web version of this article.)

Following model validation, sensitivity analysis of the tumor-bearing model was carried out to explore its multidimensional parameter space in order to assess the significance and magnitude of how changes in model parameters of interest affect NP pharmacokinetics and their delivery to the tumor. The model parameters chosen for the analysis can be classified into NP-related parameters, tumor-related parameters, and individual-related parameters (Table 3). For sensitivity analysis, we compared model outputs due to parameter perturbations to the reference model outputs shown in Fig. 3, which were obtained by simulating the model with the reference parameter values given in Table 3. For example, the whole-body pharmacokinetics for a reference NP of size 100 nm simulated under reference conditions and reference parameter values for a spherical tumor of diameter 1 cm is shown in Fig. 3a. The corresponding ${\mathrm{AUC}}_{0-\infty }$ of each compartment is shown in Fig. 3b, out of which the ${AUC}_{0-\infty }$ of the plasma compartment (${AUC}_{0-\infty }^{plasma}$), MPS super-compartment^1^ (${AUC}_{0-\infty }^{MPS}$), tumor interstitium sub-compartment (${AUC}_{0-\infty }^{tumor}$), and total excreta (${AUC}_{0-\infty }^{excreta}$) were used as the model outputs of choice for sensitivity analysis.

#### 3.2.1 Local sensitivity analysis (LSA) and parameter ranking

##### 3.2.1.1 NP-related parameters

###### 3.2.1.1.1 NP size

**Fig. 4:**
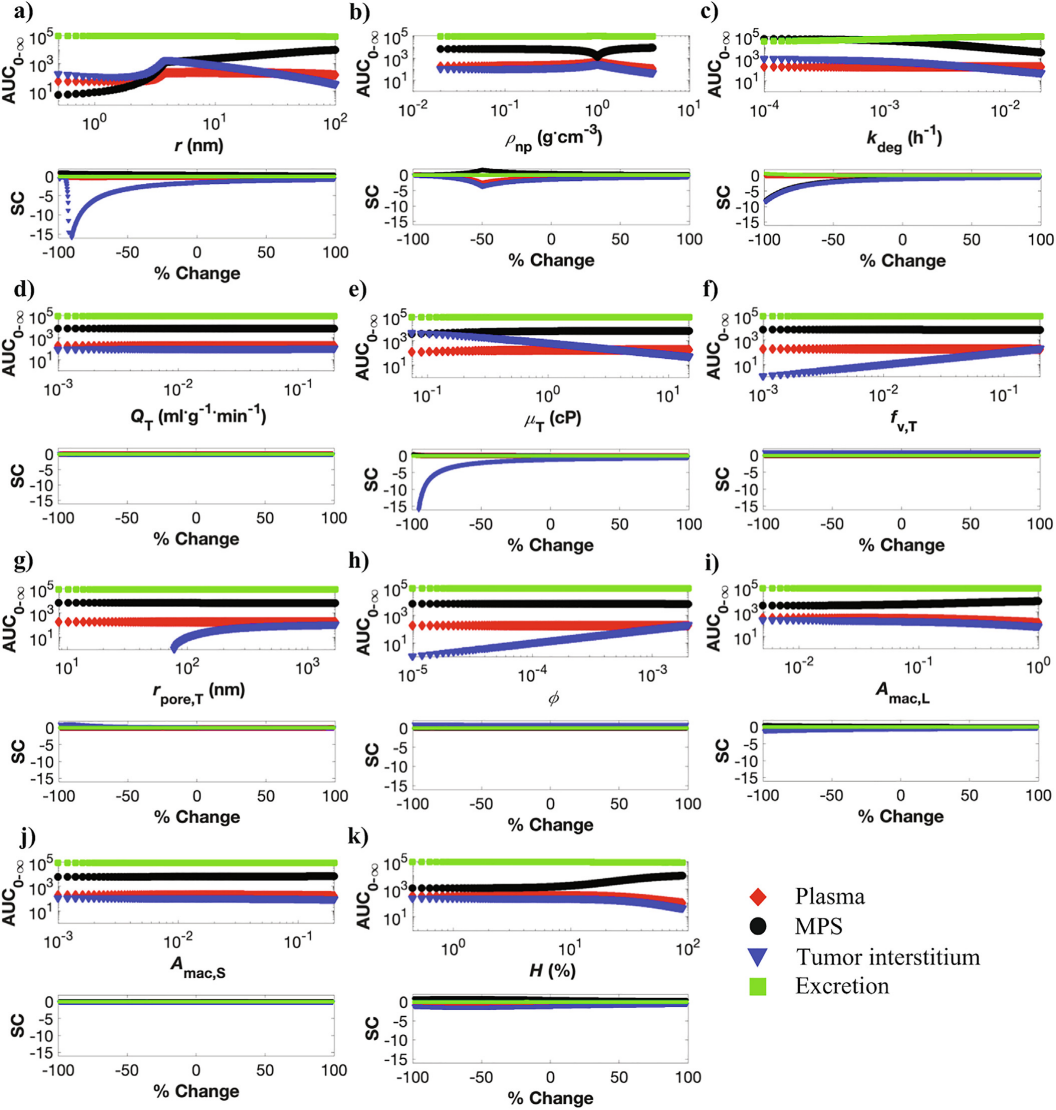
Local sensitivity analysis (LSA). Model outputs (${AUC}_{0-\infty }$, units %ID⋅h) and sensitivity coefficients (SC) following perturbation of a) NP radius, b) NP density, c) NP degradation rate, d) tumor plasma flow rate, e) tumor blood viscosity, f) tumor vascular fraction, g) tumor vascular pore radius, h) tumor vascular porosity, i) liver macrophage area fraction, j) spleen macrophage area fraction, and k) hematocrit are presented. The upper panel in each figure shows the effect of parameter perturbation on the ${AUC}_{0-\infty }$ of four model oputputs (plasma, MPS, tumor, and excreta), while the lower panel shows the corresponding SC. Each parameter was perturbed within the ±99% range around their respective literature-based mean values (Table 3).

We tested the effect of NP size in the clinically meaningful range of 1–199 nm. As shown in Fig. 4a (upper panel), at ~4 nm (equivalent to ~8 nm diameter) we see a deflection in the ${AUC}_{0-\infty }$ of plasma, MPS, and tumor interstitium compartments. This deflection point coincides with the physiological cutoff of ~8 nm for glomerular filtration (see also Fig. S4), thereby representing the point after which renal clearance of NPs becomes negligible [14]. With respect to NP accumulation in the tumor interstitium (represented by ${AUC}_{0-\infty }^{tumor}$), accumulation is highest around the deflection point, suggesting that neither very large particles nor very small (rapidly renally clearable) particles can accumulate efficiently in the tumor interstitium. As shown in Fig. S2a, to quantify the window of optimal NP size, we estimated the tumor delivery efficiency as ${AUC}_{0-t}^{tumor}/t$, where t was fixed at 1000 h (~infinity), and observed out that only NPs ranging from 7 to 22 nm diameter have a delivery efficiency >0.7% ID. (0.7% ID is the average tumor delivery efficiency across a wide variety of NPs, based on a published metanalysis of NP pharmacokinetics [3]). Therefore, consistent with other observations [16], [58], [59], NPs ranging in hydrodynamic diameter from ~7 to 22 nm seem to be able to better exploit the enhanced permeability and retention (EPR) effect-based passive accumulation in solid tumors compared to other sizes.

A similar trend was observed for the plasma compartment, although the impact of NP size on plasma bioavailability is less pronounced than its impact on tumor NP accumulation. Further, unlike plasma and tumor, NP accumulation in the MPS increases beyond the deflection point for larger particles. To distil these observations, we simulated NPs of various sizes from 1 to 199 nm (Fig. S2b), and observed that ultrasmall NPs (<7 nm dia.) are removed relatively quickly from the plasma compartment through the renal pathway, thereby minimizing their circulation time in plasma and lowering their accumulation in the MPS, tumor, or remaining organs. For larger particles (7–50 nm dia.), plasma circulation time is longer than ultrasmall NPs, primarily due to reduced renal clearance and only moderate MPS accumulation, thereby allowing efficient delivery to the tumor. Beyond 50 nm, MPS accumulation increases significantly and thus plasma circulation time goes down, thereby also lowering tumor delivery. Finally, at long term, all particle sizes are almost completely excreted, although the relative excretion through renal and hepatic routes depends on their size.

###### 3.2.1.1.2 NP density

Next, we explored the impact of NP density, which has been an underexplored parameter so far, on tumor accumulation and NP pharmacokinetics. Although, for uniformity across parameters, we perturbed the density parameter from ± 99% of its reference value (2 g⋅cm^−3^), we restrict our discussion of the density parameter to the right-hand side of the curve in Fig. 4b (>1 g⋅cm^−3^), because physically the density of NPs is usually >1 g⋅cm^−3^. As shown in Fig. 4b (upper panel), NP density > 1 g⋅cm^−3^ reduces plasma bioavailability and accumulation in tumor interstitium, apparently due to increased accumulation in the MPS, as supported by evidence in the literature [60]. Excretion remains unaffected by NP density, and all particles are completely excreted at long time. As a caveat of LSA, we can be conclusive about these results only for the reference NP size of 100 nm. To overcome this limitation, we conducted GSA to more comprehensively study the effect of parameters (Section 3.2.2).

###### 3.2.1.1.3 NP degradation rate

The *in vivo* degradation rate of NPs is a lumped parameter that accounts for NP degradation and metabolism due to the combined activity of metabolic proteins, endosomal or lysosomal enzymes, and hydrolysis. In our model, we assume that NP degradation occurs when NPs are localized either in the MPS macrophages or in the tumor interstitium, but not when particles are in circulation. Also, we assume that the rate of removal of degraded NPs from these sites into the feces is faster than the rate of degradation itself, and as a result, the rate of degradation, which is the rate limiting step, is used as a substitute for rate of excretion from MPS macrophages and the tumor interstitium.

From Fig. 4c (upper panel), it can be observed that increasing degradation rate increases total excretion and lowers NP accumulation in the MPS and tumor interstitium. It is important to understand that increased degradation rate reduces tumor or MPS accumulation not by reducing sequestration of NPs in the tumor or MPS, but by increasing the degradation of NPs accumulated in these compartments. This is corroborated by the observation that plasma kinetics remains unaltered by the degradation rate, indicating that this parameter acts downstream of the plasma compartment, i.e., it acts upon NPs that are already removed from the plasma.

##### 3.2.1.2 Tumor-related parameters

One common observation across all tumor-related parameters (Fig. 4d–h) is that they do not impact the global disposition of NPs, e.g., plasma, MPS, or excretion remain relatively unperturbed by alterations in the tumor-related parameters. Since a tumor typically comprises only a minute fraction of the organism (e.g., ~0.5 g in a 200 g rat in our simulations), it is expected that NP accumulation in the tumor does not significantly shift the mass balance of the *in vivo* system.

Surprisingly, we also observed that the tumor plasma flow rate parameter does not significantly affect tumor accumulation, at least in the studied range (Fig. 4d). To understand this observation, we simulated the model at different flow rates for two different NP sizes (10 and 200 nm). As seen in Fig. S3, tumor accumulation showed only minimal variation across flow rates for a given NP size, although it did vary significantly between particles sizes. The corresponding plasma concentrations in Fig. S3 also vary across NP sizes but not across tumor plasma flow rates, indicating that plasma concentration corrleates directly with tumor accumulation, and the impact of plasma concentration is much stronger than tumor plasma flow rate in governing the accumulation in the tumor interstitium. Therefore, NPs with longer plasma half-lives can accumulate more in the tumor interstitium relative to NPs with shorter plasma half-lives, irrespective of tumor plasma flow rates.

As is already known, tumor blood viscosity is generally higher in tumors than in healthy tissues [61], which as seen in Fig. 4e can reduce the accumulation of NPs in tumor interstitium, arguably due to reduction in the diffusivity of NPs that can reduce the permeability parameter (Eq. (7)) and lower transvascular migration across porous tumor vessel walls. Further, tumor vascular fraction (Fig. 4f), tumor vascular pore size (Fig. 4g), and tumor vascular porosity (Fig. 4h) are directly correlated to tumor intersitium accumulation of NPs. As we know from Eqs. (7), (S36), and section 3 of SI, all three parameters are directly related to the permeability-surface area product of the tumor, which governs the diffusion-dependent rate of NP influx into the tumor interstitium, thereby explaining the behavior of these parameters.

##### 3.2.1.3 Individual-related parameters

We further investigated parameters that have been experimentally tuned to improve the efficacy of nanomedicine (e.g. macrophage reduction [12]), or can potentially change in a cancer-bearing patient (e.g. hematocrit [61]). As shown in Fig. 4i, reduction in Kupffer cell concentration in the liver can reduce MPS accumulation and thus increase plasma bioavailability or accumulation in the tumor interstitium, consistent with other observations in the literature [12]. Reduction in splenic macrophages has a similar effect, but the impact is not as pronounced, most likely due to the lesser supply of NPs to the spleen compared to the liver (based on the difference in the mass of NPs supplied via plasma flow) (Fig. 4j). Finally, hematocrit, which directly affects erythrocyte-enhanced diffusion of NPs (Eq. (2)) if elevated in the systemic circulation, can reduce the circulation half-life of NPs in the plasma compartment due to the enhancement of the $k_{on,i}$ parameter (Eq. (3)). Increase in $k_{on,i}$ will increase the deposition of NPs on the microvascular walls of the organs, which will especially benefit NP sequestration in the macrophages in the MPS, thereby causing increased accumulation in the these organs, as seen in Fig. 4k. As a result of reduction of systemic concentration of NPs, the delivery efficiency to the tumor interstitium will decrease.

##### 3.2.1.4 LSA-based parameter ranking

**Fig. 5:**
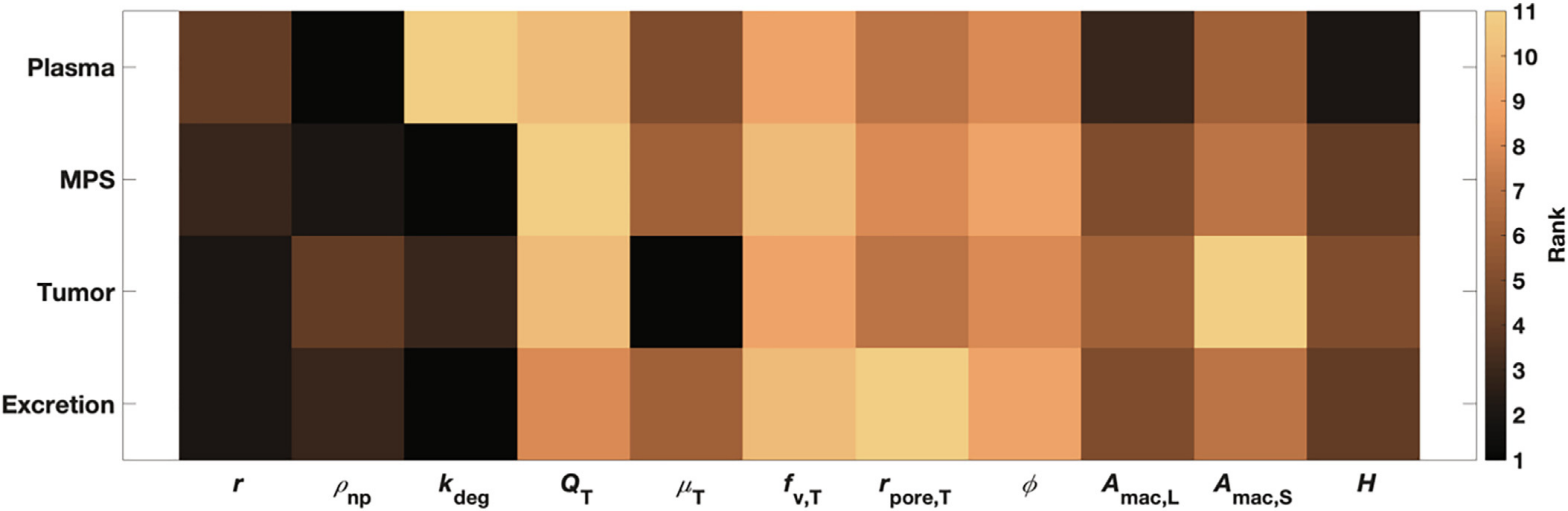
Parameter ranking from local sensitivity analysis. Note: Rank 1 denotes parameter of the highest significance, and rank 11 of the lowest.

Parameter ranking was obtained based on the findings of the LSA, which shows that tumor accumulation is most strongly affected by blood viscosity, followed by NP size and degradation rate (Fig. 5). Plasma bioavailability is most affected by NP density, followed by hematocrit and Kupffer cell density. NP accumulation in the MPS depends mostly on NP-related parameters, with degradation rate found to be the most important parameter, followed by NP density and size. Finally, as expected, excretion is primarily affected by NP degradation rate.

#### 3.2.2 Global sensitivity analysis (GSA) and parameter ranking

**Fig. 6:**
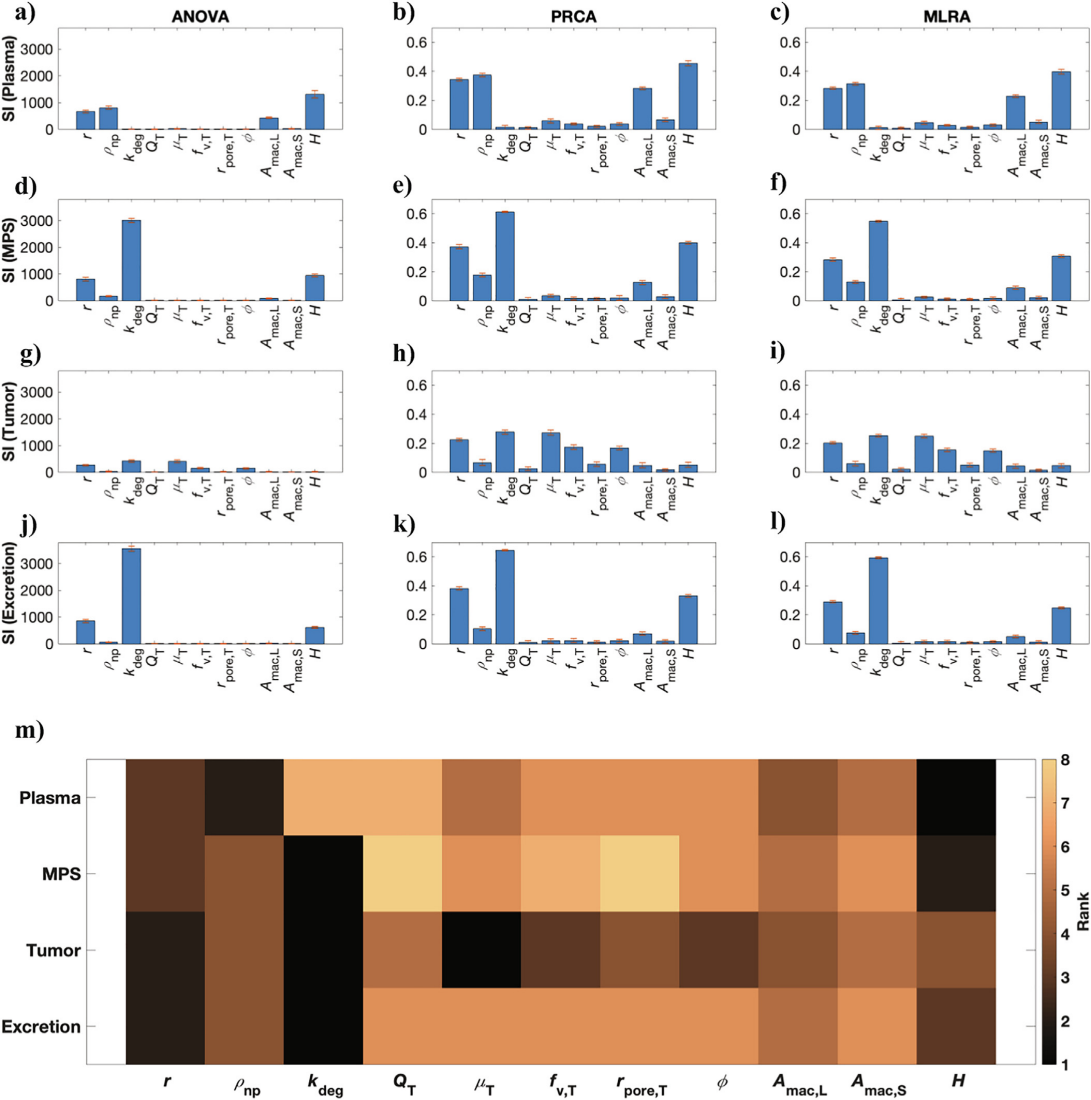
Global sensitivity analysis (GSA). Results of GSA from ANOVA (a, d, g, j), PRCA (b, e, h, k), and MLRA (c, f, i, l) are shown. m) Weighted parameter ranking as obtained from the weighted average of rankings obtained from the above three techniques. Note: Rank 1 denotes parameter of the highest significance, and rank 8 of the lowest.

Lastly, we conducted GSA to overcome the limitations of LSA and explore the impact of simultaneous parameter perturbations on tumor delivery and whole-body pharmacokinetics of NPs. As shown in Fig. 6, all three GSA techniques (ANOVA, PRCA, MLRA) confirm the finding from LSA that tumor-related parameters do not significantly affect the plasma bioavailability (Fig. 6a–c), MPS accumulation (Fig. 6d–f), or excretion (Fig. 6j–l) of NPs. This further supports the argument that the presence of a tumor does not significantly affect the mass balance of the system given the premise that a very small amount (<1% ID) of NPs ever reaches the tumors. However, tumor size, which was not investigated here, may have an impact such that larger tumors may accumulate enough mass to affect the systemic kinetics of NPs.

With respect to the plasma bioavailability of NPs, as shown in Fig. 6a–c, GSA shows that hematocrit is the most critical parameter, followed by NP density, NP size, and Kupffer cell area fraction. As shown in Fig. 5, Fig. 6m, while GSA and LSA both rank these four parameters as highly significant for plasma bioavailability, the ranking order obtained is slightly different depending on which sensitivity analysis method is used. Thus, the most easily tunable parameters, i.e. NP size and NP density, are critical in affecting the systemic bioavailability of NPs, with particles in the diameter range of 7–22 nm and with density close to the density of plasma (e.g. liposomes) being ideal candidates for applications that demand long term systemic circulation of NPs.

Further, in the context of MPS accumulation (Fig. 6d–f), NP degradation was found to be the most critical parameter. However, as discussed before, this only indicates that high NP degradation rates lead to lesser accumulation in the MPS due to faster excretion of NPs from MPS, rather than due to reduced uptake from the systemic blood. Thus, this tunable parameter can be of significance in the context of the potential hepatotoxic effects of some nanomaterials. With respect to parameters that affect the uptake of NPs by the MPS, hematocrit followed by NP size and NP density are of the greatest importance. This observation correlates with the observation for the plasma compartment, indicating that NPs with optimal size and density attain higher systemic bioavailability due to reduced MPS accumulation of NPs. Interestingly, the macrophage area density parameter ranks very low in the GSA ranking (Fig. 6m), indicating that depletion of macrophages does not provide for an effective strategy to reduce MPS accumulation of NPs. Further, as we will see in the case of tumor delivery of NPs, macrophage area fraction does not seem to be a critical parameter either, as also supported by evidence in the literature [12]. These results indicate that the sinusoidal fenestrations and other cells in the MPS should be further investigated to better understand their systematic role in MPS accumulation of NPs.

In the context of delivery to the tumor interstitium, GSA upholds the importance of tumor blood viscosity, as predicted by LSA, but considers NP degradation rate to be equally important. The role of NP degradation rate in the tumor is similar to the degradation rate in the MPS, and thus NP concentration in the tumor is reduced due to NP degradation (as opposed to limited NP delivery), analogous to what was observed in the MPS compartments. Importantly, NP size, tumor vascular porosity, and tumor vascular fraction are critical parameters in governing tumor accumulation of NPs, arguably due to their ability to affect the P∙S parameter (Eq. (7)). While hematocrit is critical in affecting plasma bioavailability and MPS accumulation, it seems relatively less important for tumor accumulation of NPs. Finally, as expected, NP degradation rate is the most relevant parameter concerning NP excretion, as was also suggested by LSA.

While other NP physicochemical properties such as polydispersity, surface charge, and surface chemistry are considered critical in governing the pharmacokinetics of NPs, they are out of the scope of the current model and will be considered in future investigations. As discussed before, the drug loading capacity of NPs and the IC_50_ of the payload are important parameters when considering the pharmacodynamics of drugs delivered through NPs; however our focus in this study is on the systemic disposition of NPs, and as such these parameters are not included in the current investigation. Also, becuase spatial dependency is homogenized through integration over space in a PBPK model, such that bivariate components (space and time) are reduced to time-dependent averages [62], it becomes challenging to incorporate spatial heterogeneities in tumor vascularization and extracellular matrix density that affect intratumoral distribution of nanomaterials and drugs [63]. Thus, our assumption of a homogeneous tumor compartment means our model is unable to provide information on the spatial distribution of NPs in the tumor. In the future, we will consider a more detailed tumor compartment through introduction of a discrete agent-based tumor description in our model. Finally, inter-animal variation in physiological parameters (due to differences in age, gender, and breed) that were assumed to be constant in the current study may be a source of variation in the pharmacokinetics and tumor deliverability of NPs, which will be explored in future investigations.

## 4 Conclusions

We developed a generalized PBPK model to study the whole-body pharmacokinetics of NPs and to investigate their tumor deliverability. The model, based on physiological parameters that were known a priori or derived mechanistically, can be used in its current form to predict the *in vivo* behavior of NPs, in particular the plasma circulation time, MPS accumulation, excretion kinetics, and tumor delivery efficiency, solely based on the NP size, density, and degradation rate. It can also be used to simulate physiological or pathophysiological conditions to explore unknown scenarios for investigating tumor delivery efficiency of nanomedicine, and thus generate NP design guidelines or personalized treatment strategies. LSA and GSA revealed the importance of NP degradation rate, tumor blood viscosity, NP size, tumor vascular fraction, and tumor vascular porosity in affecting delivery to the tumor interstitium. In these analyses, we did not include parameters that are practically constant irrespective of the presence of the tumor. Thus, only parameters that are tunable, or that change due to the presence of the tumor, or belong to the tumor compartment itself were included. Following appropriate inter-species scaling, our predictive modeling platform holds the key to test *in silico* the efficacy of cancer nanomedicine in a variety of imaginable clinical scenarios.

## Author contributions

ZW conceived and supervised the research. PD and ZW designed the research. PD developed the mathematical model. PD, JDB, JRR, YLC collected the data and performed the model analysis. PD, JDB, JRR, YLC, AN, CJB, VC, ZW interpreted the data and model results. PD, JDB, JRR, YLC, AN, CJB, VC, ZW wrote the manuscript.

## Declaration of Competing Interest

The authors declare that they have no known competing financial interests or personal relationships that could have appeared to influence the work reported in this paper.

## Footnotes

1 MPS super-compartment includes both liver and spleen.
