# Supplementary material for: A mathematical model to predict nanomedicine pharmacokinetics and tumor delivery
Source: Comput Struct Biotechnol J. 2020 Feb 29;18:518–31. doi: 10.1016/j.csbj.2020.02.014 (PMC7078505; doi:10.1016/j.csbj.2020.02.014)
Supplement: Supplementary data 1 [file mmc1.docx]

**Supplementary Information**

**A mathematical model for nanomedicine pharmacokinetics and tumor delivery**

Prashant Dogra, Joseph D. Butner, Javier Ruiz Ramírez, Yao-li Chuang, Achraf Noureddine, C. Jeffrey Brinker, Vittorio Cristini, Zhihui Wang

**Supplementary Figures**


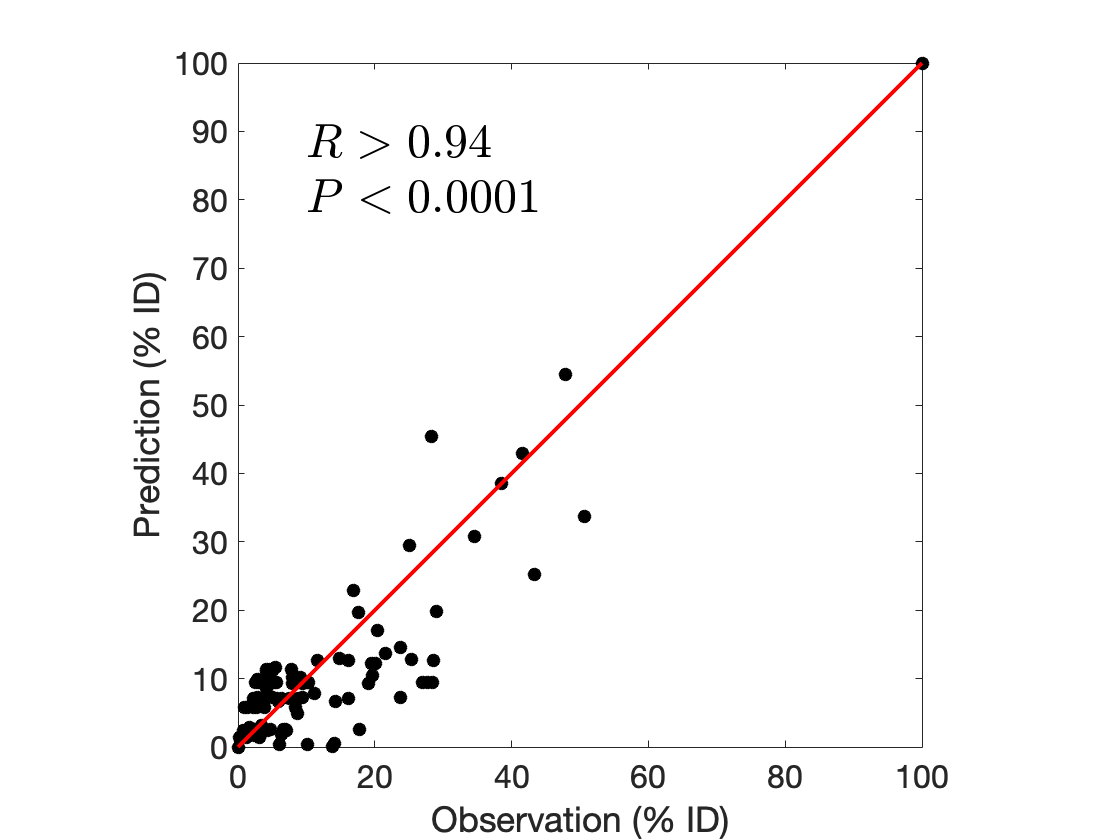


**Figure S1. Goodness of predictive performance of the model.** Pearson correlation between experimental observations and model predictions (pooled data and model outputs of all four NP sizes) show strong agreement as indicated by the correlation coefficient value > 0.94. Red line denotes the $y=x$ line.


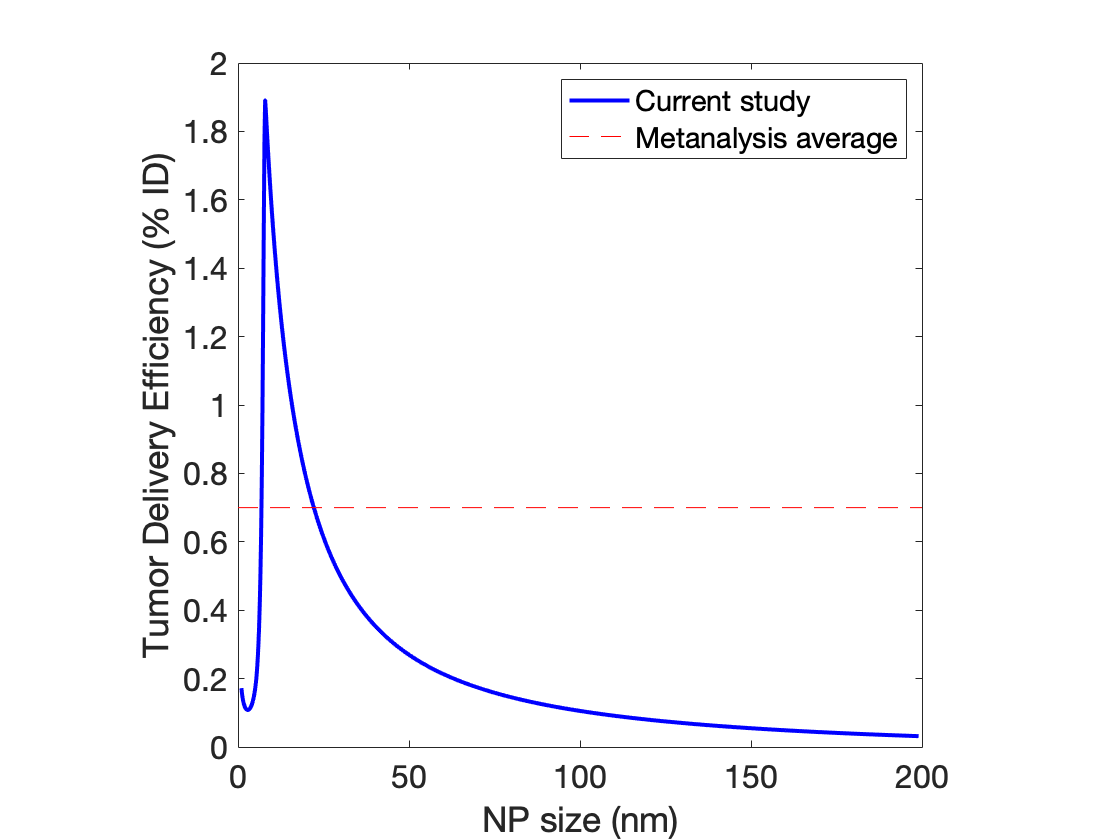


**a)**


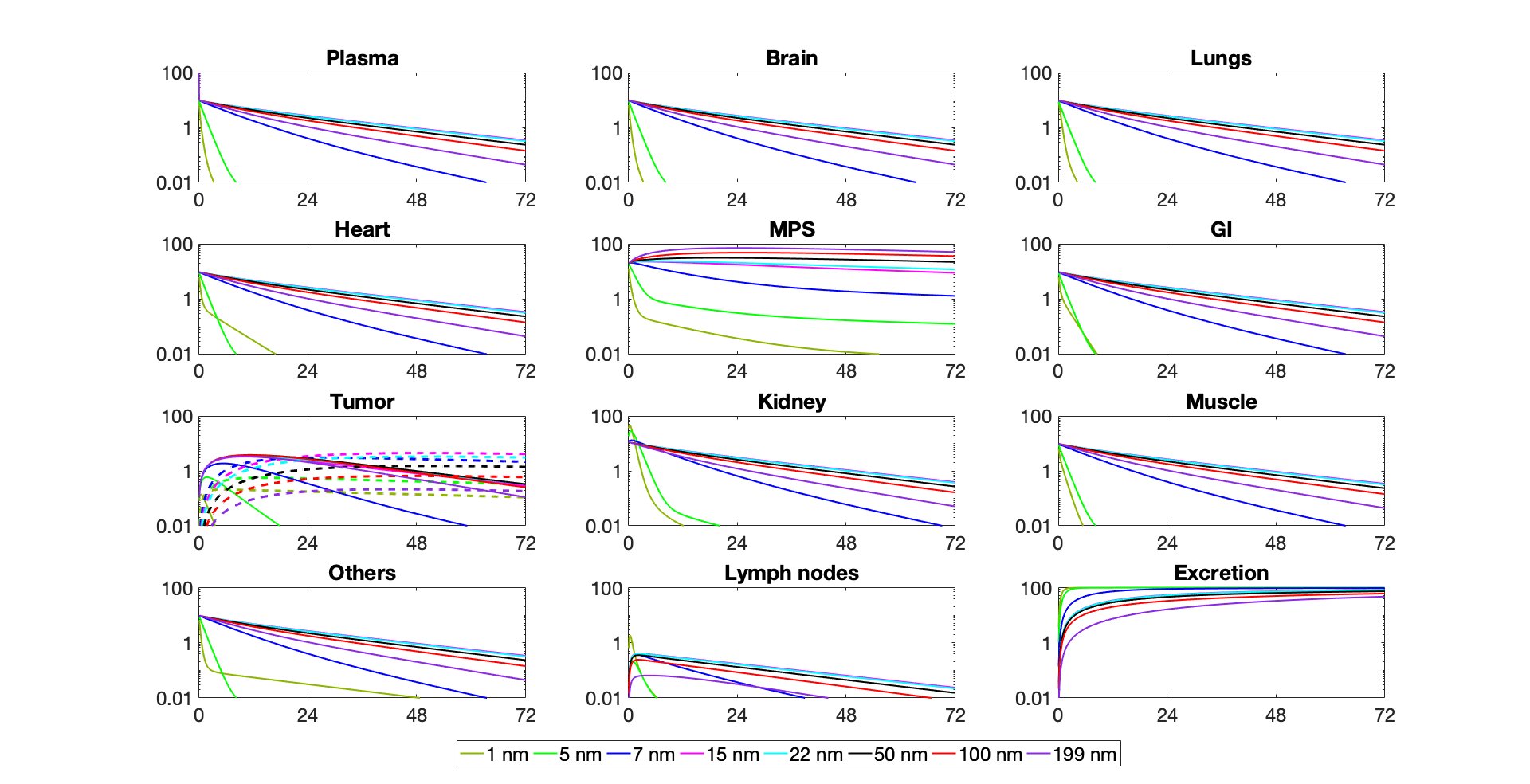


**Time (h)**

**% ID**

**b)**

**Figure S2. Effect of NP size. a)** NPs within the size range of 7-22 nm dia. have a tumor delivery efficiency greater than the accepted average tumor delivery efficiency reported across the clinically-relevant NP size range (i.e. 0.7% ID; red dashed line). **b)** Effect of NP size (dia.) on whole-body disposition and tumor delivery is shown. Dashed lines and solid lines in the tumor panel represent NP mass kinetics in the tumor interstitium and tumor vascular space, respectively. Solid lines in the remaining panels denote the total NP mass kinetics in the given compartment.

**a) 10 nm**

**b) 200 nm**


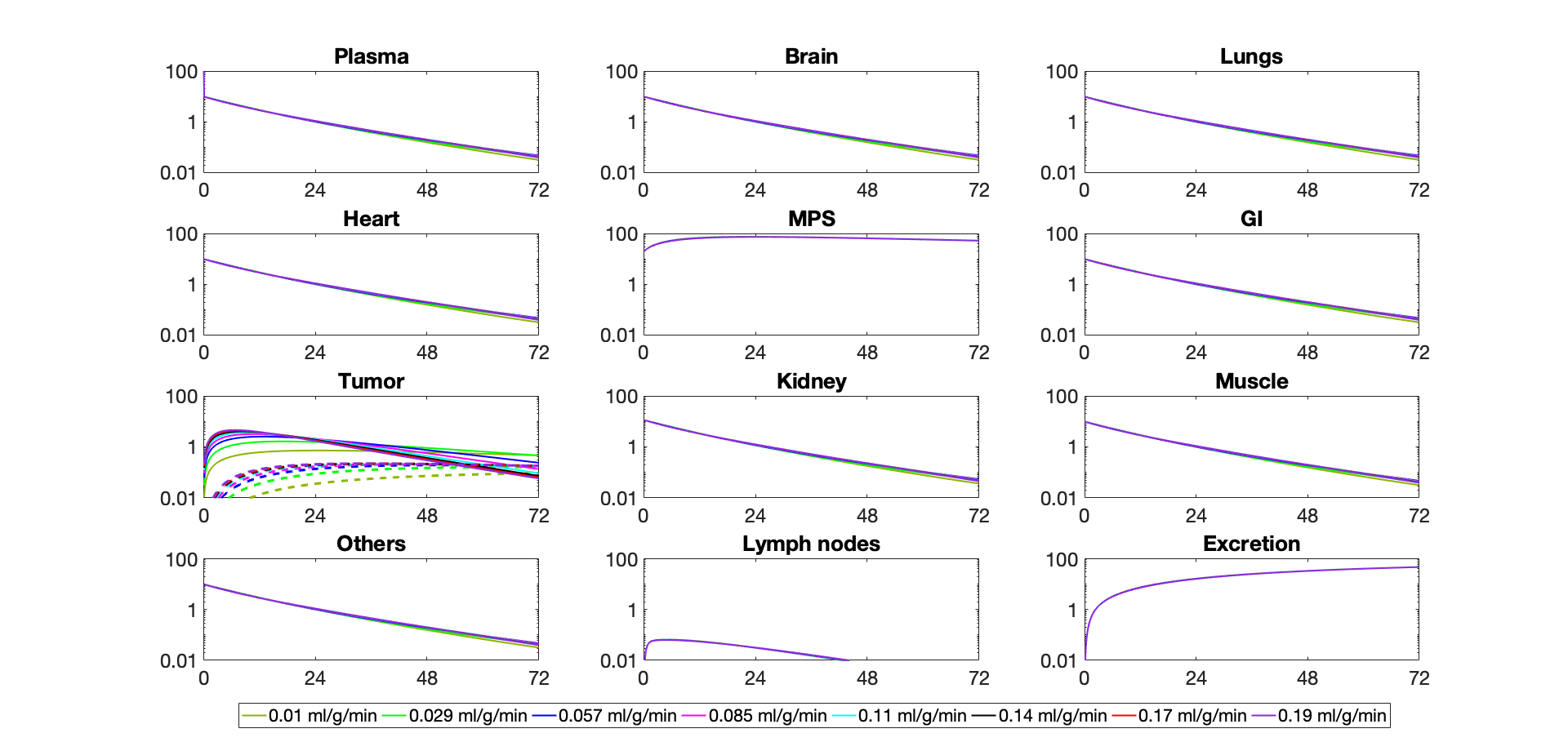


**Time (h)**

**% ID**


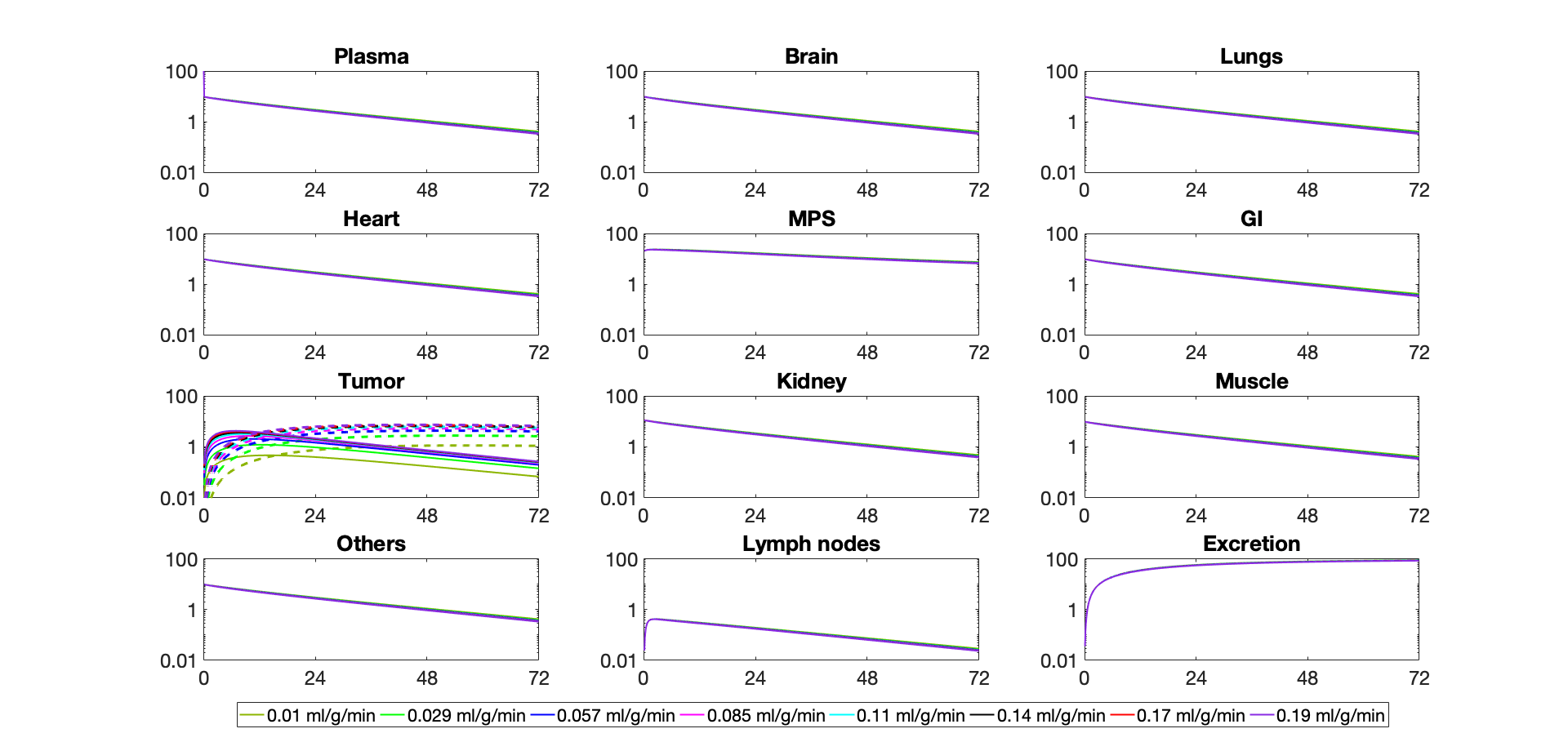


**Time (h)**

**% ID**

**Figure S3. Effect of tumor blood flow rate.** Model was simulated for NP diameters of **a)** 10 nm and **b)** 200 nm with different plasma flow rate values to study the impact of flow rate on tumor accumulation of NPs. Dashed lines and solid lines in the tumor panel represent NP mass kinetics in the tumor interstitium and tumor vascular space, respectively. Solid lines in the remaining panels denote the total NP mass kinetics in the given compartment.

**b)**


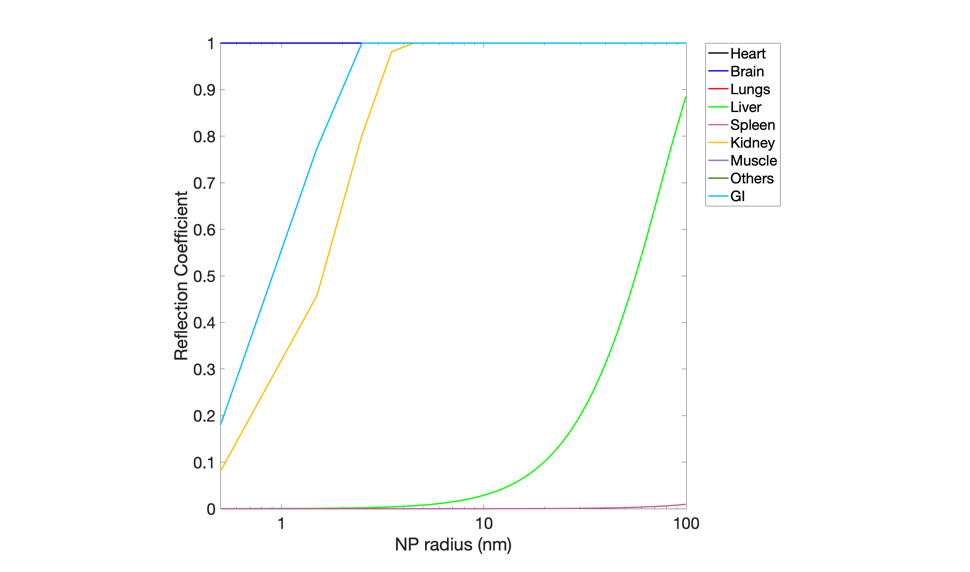


**a)**


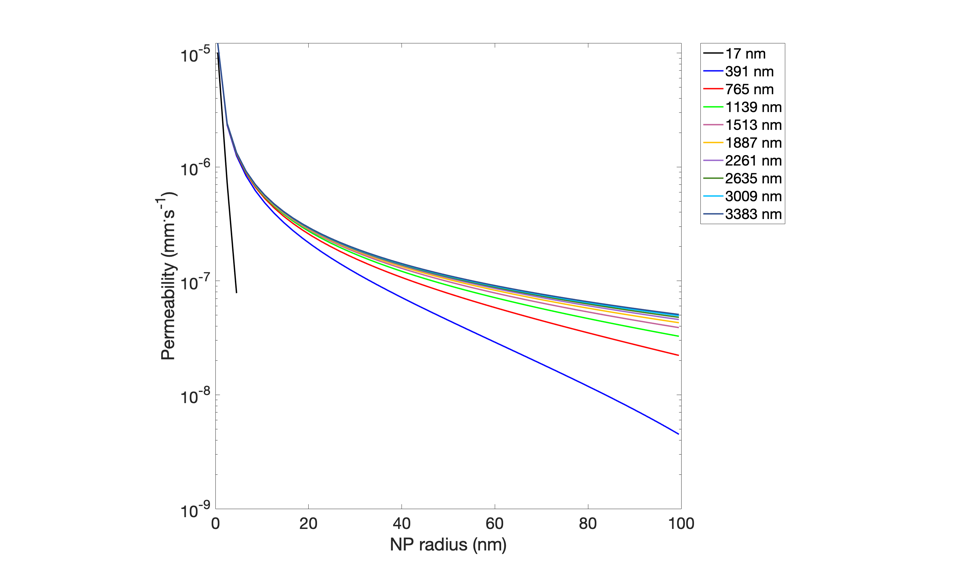


**Figure S4. Microvascular extravasation parameters as a function of NP radius. a)** Variation in vascular reflection coefficient is shown for different organs. Note: since the radius of vascular fenestrations in lungs, heart, GI, muscle, and others is chosen to be the same (2.5 nm), their reflection coefficient curves overlap (cyan color). **b)** Variation in vascular permeability is shown for different vascular pore diameters of a tumor (ranging between ± 99% of 1700 nm).

**Supplementary Methods**

1. **PBPK model equations**

The PBPK model is based on conservation of mass and the law of mass action. The complete system of equations (S1-S35), with the initial conditions, is described as follows (refer to Figure 1b for the model structure).

- 1. **Brain compartment**

1. **Vascular sub-compartment**

*Free NPs*

$V_{v,B}\frac{dC_{v,B}^{f}}{dt}={Q_{B}C}_{P}-\left( Q_{B}-L_{B} \right)C_{v,B}^{f}-{L_{B}\cdot\left( 1-\sigma_{B} \right)C}_{v,B}^{f}-{k_{on,B}V_{v,B}C}_{v,B}^{f}+{k_{off,B}V_{v,B}C}_{v,B}^{b}$,

$V_{v,B}C_{v,B}^{f}\left( 0 \right)=0$ (S1)

*Bound NPs*

$V_{v,B}\frac{dC_{v,B}^{b}}{dt}={k_{on,B}V_{v,B}C}_{v,B}^{f}-{k_{off,B}V_{v,B}C}_{v,B}^{b}$, $V_{v,B}C_{v,B}^{b}\left( 0 \right)=0$ (S2)

where $C_{v,i}^{f}$ represents the concentration of free NPs in the vascular sub-compartment of organ $i$, such that the subscript $i=B$ for brain, $i=H$ for heart, $i=LU$ for lungs, $i=L$ for liver, $i=K$ for kidney, $i=M$ for muscle, $i=O$ for others, $i=T$ for tumor, $i=S$ for spleen, and $i=\mathrm{GI}$ for gastrointestinal tract. $V_{v,i}$ represents the volume of the vascular sub-compartment of organ $i$; $C_{P}$ represents the plasma concentration of NPs; $Q_{i}$ and $L_{i}$ are the plasma and lymph flow rates of organ $i$, respectively; $\sigma_{i}$ is the vascular reflection coefficient of organ $i$; $k_{on,i}$ and $k_{off,i}$ are the rates of NP binding to the vascular wall and dislodging from the vascular wall, respectively; and $C_{v,i}^{b}$ is the concentration of bound NPs in the vascular sub-compartment of organ $i$.

1. **Extravascular sub-compartment**

$V_{e,B}\frac{dC_{e,B}}{dt}={L_{B}\cdot\left( 1-\sigma_{B} \right)C}_{v,B}^{f}-{L_{B}C}_{e,B}$, $V_{e,B}C_{e,B}\left( 0 \right)=0$ (S3)

where $C_{e,i}$ is the concentration of NPs in the extravascular sub-compartment of organ $i$, which has an extravascular volume $V_{e,i}$.

- 1. **Heart compartment**

**(a) Vascular sub-compartment**

*Free NPs*

$V_{v,H}\frac{dC_{v,H}^{f}}{dt}={Q_{H}C}_{P}-\left( Q_{H}-L_{H} \right)C_{v,H}^{f}-{L_{H}\cdot\left( 1-\sigma_{H} \right)C}_{v,H}^{f}-{k_{on,H}V_{v,H}C}_{v,H}^{f}+{k_{off,H}V_{v,H}C}_{v,H}^{b}$,

$V_{v,H}C_{v,H}^{f}\left( 0 \right)=0$ (S4)

*Bound NPs*

$V_{v,H}\frac{dC_{v,H}^{b}}{dt}={k_{on,H}V_{v,H}C}_{v,H}^{f}-{k_{off,H}V_{v,H}C}_{v,H}^{b}$, ${V_{v,H}C}_{v,H}^{b}\left( 0 \right)=0$ (S5)

1. **Extravascular sub-compartment**

$V_{e,H}\frac{dC_{e,H}}{dt}={L_{H}\cdot\left( 1-\sigma_{H} \right)C}_{v,H}^{f}-{L_{H}C}_{e,H}$, ${V_{e,H}C}_{e,H}\left( 0 \right)=0$ (S6)

- 1. **Lungs compartment**

**(a) Vascular sub-compartment**

*Free NPs*

$V_{v,LU}\frac{dC_{v,LU}^{f}}{dt}={Q_{\mathrm{LU}}C}_{P}-\left( Q_{\mathrm{LU}}-L_{\mathrm{LU}} \right)C_{v,LU}^{f}-{L_{\mathrm{LU}}\cdot\left( 1-\sigma_{\mathrm{LU}} \right)C}_{v,LU}^{f}-{k_{on,LU}V_{v,LU}C}_{v,LU}^{f}+{k_{off,LU}V_{v,LU}C}_{v,LU}^{b}$,

${V_{v,LU}C}_{v,LU}^{f}\left( 0 \right)=0$ (S7)

*Bound NPs*

$V_{v,LU}\frac{dC_{v,LU}^{b}}{dt}={k_{on,LU}V_{v,LU}C}_{v,LU}^{f}-{k_{off,LU}V_{v,LU}C}_{v,LU}^{b}$, $V_{v,LU}C_{v,LU}^{b}\left( 0 \right)=0$ (S8)

1. **Extravascular sub-compartment**

$V_{e,LU}\frac{dC_{e,LU}}{dt}={L_{\mathrm{LU}}\cdot\left( 1-\sigma_{\mathrm{LU}} \right)C}_{v,LU}^{f}-{L_{\mathrm{LU}}C}_{e,LU}$, $V_{e,LU}C_{e,LU}\left( 0 \right)=0$ (S9)

- 1. **Spleen compartment**

**(a) Vascular sub-compartment**

*Free NPs*

$V_{v,S}\frac{dC_{v,S}^{f}}{dt}={Q_{S}C}_{P}-\left( Q_{S}-L_{S} \right)C_{v,S}^{f}-{L_{S}\cdot\left( 1-\sigma_{S} \right)C}_{v,S}^{f}-{k_{on,S}V_{v,S}C}_{v,S}^{f}+{k_{off,S}V_{v,S}C}_{v,S}^{b}$,

$V_{v,S}C_{v,S}^{f}\left( 0 \right)=0$ (S10)

*Bound NPs*

$V_{v,S}\frac{dC_{v,S}^{b}}{dt}={k_{on,S}V_{v,S}C}_{v,S}^{f}-{k_{off,S}V_{v,S}C}_{v,S}^{b}-{k_{\mathrm{mac}}A_{mac,S}V_{v,S}C}_{v,S}^{b}$,

${V_{v,S}C}_{v,S}^{b}\left( 0 \right)=0$ (S11)

where $A_{mac,i}$ represents the area fraction of macrophages in the microvasculature of organ $i$ and $k_{\mathrm{mac}}$ is the rate of NP phagocytosis by endothelium-lining macrophages.

1. **Extravascular sub-compartment**

$V_{e,S}\frac{dC_{e,S}}{dt}={L_{S}\cdot\left( 1-\sigma_{S} \right)C}_{v,S}^{f}-{L_{S}C}_{e,S}$, ${V_{e,S}C}_{e,S}\left( 0 \right)=0$ (S12)

1. **Phagocytic sub-compartment**

$\frac{dN_{p,S}}{dt}={k_{\mathrm{mac}}A_{mac,S}V_{v,S}C}_{v,S}^{b}-{k_{\deg}N}_{p,S}$, $N_{p,S}\left( 0 \right)=0$ (S13)

where $N_{p,i}$ is the mass of phagocytized NPs; $k_{\deg}$ is the rate of NP degradation (and excretion).

- 1. **Gastrointestinal tract (GI) compartment**

**(a) Vascular sub-compartment**

*Free NPs*

$V_{v,GI}\frac{dC_{v,GI}^{f}}{dt}={Q_{\mathrm{GI}}C}_{P}-\left( Q_{\mathrm{GI}}-L_{\mathrm{GI}} \right)C_{v,GI}^{f}-{L_{\mathrm{GI}}\cdot\left( 1-\sigma_{\mathrm{GI}} \right)C}_{v,GI}^{f}-{k_{on,GI}V_{v,GI}C}_{v,GI}^{f}+{k_{off,GI}V_{v,GI}C}_{v,GI}^{b}$,

${V_{v,GI}C}_{v,GI}^{f}\left( 0 \right)=0$ (S14)

*Bound NPs*

$V_{v,GI}\frac{dC_{v,GI}^{b}}{dt}={k_{on,GI}V_{v,GI}C}_{v,GI}^{f}-{k_{off,GI}V_{v,GI}C}_{v,GI}^{b}$, ${V_{v,GI}C}_{v,GI}^{b}\left( 0 \right)=0$ (S15)

1. **Extravascular sub-compartment**

$V_{e,GI}\frac{dC_{e,GI}}{dt}={L_{\mathrm{GI}}\cdot\left( 1-\sigma_{\mathrm{GI}} \right)C}_{v,GI}^{f}-{L_{G}C}_{e,GI}$, $V_{e,GI}C_{e,GI}\left( 0 \right)=0$ (S16)

- 1. **Liver compartment**

**(a) Vascular sub-compartment**

*Free NPs*

$V_{v,L}\frac{dC_{v,L}^{f}}{dt}={Q_{\mathrm{HA}}C}_{P}+\left( Q_{S}-L_{S} \right)C_{v,S}^{f}+\left( Q_{\mathrm{GI}}-L_{\mathrm{GI}} \right)C_{v,GI}^{f}-\left( Q_{L}-L_{L} \right)C_{v,L}^{f}-{L_{L}\cdot\left( 1-\sigma_{L} \right)C}_{v,L}^{f}-{k_{on,L}V_{v,L}C}_{v,L}^{f}+{k_{off,L}V_{v,L}C}_{v,L}^{b}$,

$V_{v,L}C_{v,L}^{f}\left( 0 \right)=0$ (S17)

where $Q_{L}=Q_{\mathrm{HA}}+(Q_{S}-L_{S})+(Q_{\mathrm{GI}}-L_{\mathrm{GI}})$ is the total plasma flow rate into the liver; $Q_{\mathrm{HA}}$ is the contribution of plasma flow rate into the liver from the hepatic artery.

*Bound NPs*

$V_{v,L}\frac{dC_{v,L}^{b}}{dt}={k_{on,L}V_{v,L}C}_{v,L}^{f}-{k_{off,L}V_{v,L}C}_{v,L}^{b}-{k_{\mathrm{mac}}A_{mac,L}V_{v,L}C}_{v,L}^{b}$,

${V_{v,L}C}_{v,L}^{b}\left( 0 \right)=0$ (S18)

1. **Extravascular sub-compartment**

$V_{e,L}\frac{dC_{e,L}}{dt}={L_{L}\cdot\left( 1-\sigma_{L} \right)C}_{v,L}^{f}-{L_{L}C}_{e,L}-B\cdot C_{e,L}$, $V_{e,L}C_{e,L}\left( 0 \right)=0$ (S19)

where $B$ represents the bile flow rate.

**(c) Phagocytic sub-compartment**

$\frac{dN_{p,L}}{dt}={k_{\mathrm{mac}}A_{mac,L}V_{v,L}C}_{v,L}^{b}-{k_{\deg}N}_{p,L}$, $N_{p,L}\left( 0 \right)=0$ (S20)

- 1. **Kidney compartment**

**(a) Vascular sub-compartment**

*Free NPs*

$V_{v,K}\frac{dC_{v,K}^{f}}{dt}={Q_{K}C}_{P}-\left( Q_{K}-\mathrm{GFR} \right)C_{v,K}^{f}-{\mathrm{GFR}\cdot\left( 1-\sigma_{K} \right)C}_{v,K}^{f}-{k_{on,K}V_{v,K}C}_{v,K}^{f}+{k_{off,K}V_{v,K}C}_{v,K}^{b}$,

${V_{v,K}C}_{v,K}^{f}\left( 0 \right)=0$ (S21)

where $\mathrm{GFR}$ represents the glomerular filtration rate.

*Bound NPs*

$V_{v,K}\frac{dC_{v,K}^{b}}{dt}={k_{on,K}V_{v,K}C}_{v,K}^{f}-{k_{off,K}V_{v,K}C}_{v,K}^{b}$, ${V_{v,K}C}_{v,K}^{b}\left( 0 \right)=0$ (S22)

1. **Extravascular sub-compartment**

$V_{e,K}\frac{dC_{e,K}}{dt}={\mathrm{GFR}\cdot\left( 1-\sigma_{K} \right)C}_{v,K}^{f}-{U\cdot C}_{e,K}$, ${V_{e,K}C}_{e,K}\left( 0 \right)=0$ (S23)

where $U$ is the urine formation rate.

- 1. **Muscle compartment**

**(a) Vascular sub-compartment**

*Free NPs*

$V_{v,M}\frac{dC_{v,M}^{f}}{dt}={Q_{M}C}_{P}-\left( Q_{M}-L_{M} \right)C_{v,M}^{f}-{L_{M}\cdot\left( 1-\sigma_{M} \right)C}_{v,M}^{f}-{k_{on,M}V_{v,M}C}_{v,M}^{f}+{k_{off,M}V_{v,M}C}_{v,M}^{b}$,

${V_{v,M}C}_{v,M}^{f}\left( 0 \right)=0$ (S24)

*Bound NPs*

$V_{v,M}\frac{dC_{v,M}^{b}}{dt}={k_{on,M}V_{v,M}C}_{v,M}^{f}-{k_{off,M}V_{v,M}C}_{v,M}^{b}$, ${V_{v,M}C}_{v,M}^{b}\left( 0 \right)=0$ (S25)

1. **Extravascular sub-compartment**

$V_{e,M}\frac{dC_{e,M}}{dt}={L_{M}\cdot\left( 1-\sigma_{M} \right)C}_{v,M}^{f}-{L_{M}C}_{e,M}$, ${V_{e,M}C}_{e,M}\left( 0 \right)=0$ (S26)

- 1. **Others compartment**

**(a) Vascular sub-compartment**

*Free NPs*

$V_{v,O}\frac{dC_{v,O}^{f}}{dt}={Q_{O}C}_{P}-\left( Q_{O}-L_{O} \right)C_{v,O}^{f}-{L_{O}\cdot\left( 1-\sigma_{O} \right)C}_{v,O}^{f}-{k_{on,O}V_{v,O}C}_{v,O}^{f}+{k_{off,O}V_{v,O}C}_{v,O}^{b}$,

${V_{v,O}C}_{v,O}^{f}\left( 0 \right)=0$ (S27)

*Bound NPs*

$V_{v,O}\frac{dC_{v,O}^{b}}{dt}={k_{on,O}V_{v,O}C}_{v,O}^{f}-{k_{off,O}V_{v,O}C}_{v,O}^{b}$, ${V_{v,O}C}_{v,O}^{b}\left( 0 \right)=0$ (S28)

1. **Extravascular sub-compartment**

$V_{e,O}\frac{dC_{e,O}}{dt}={L_{O}\cdot\left( 1-\sigma_{O} \right)C}_{v,O}^{f}-{L_{O}C}_{e,O}$, ${V_{e,O}C}_{e,O}\left( 0 \right)=0$ (S29)

- 1. **Tumor compartment**

**(a) Vascular sub-compartment**

*Free NPs*

$V_{v,T}\frac{dC_{v,T}^{f}}{dt}={Q_{T}\cdot(C}_{P}-C_{v,T}^{f})-{P\cdot S\cdot C}_{v,T}^{f}-{k_{on,T}V_{v,T}C}_{v,T}^{f}+{k_{off,T}V_{v,T}C}_{v,T}^{b}$,

${V_{v,T}C}_{v,T}^{f}\left( 0 \right)=0$ (S30)

where $P$ is the permeability of tumor vasculature; and $S$ is the total tumor microvascular surface area.

*Bound NPs*

$V_{v,T}\frac{dC_{v,T}^{b}}{dt}={k_{on,T}V_{v,T}C}_{v,T}^{f}-{k_{off,T}V_{v,T}C}_{v,T}^{b}$, ${V_{v,T}C}_{v,T}^{b}\left( 0 \right)=0$ (S31)

**(b) Extravascular (tumor interstitium) sub-compartment**

$\frac{dN_{e,T}}{dt}={P\cdot S\cdot C}_{v,T}^{f}-{k_{\deg}N}_{e,T}$, $N_{e,T}\left( 0 \right)=0$ (S32)

where $N_{e,T}$ is the mass of NPs in the tumor interstitium.

- 1. **Lymph node compartment**

The lymph node compartment represents a lumped compartment that contains all the lymph nodes of an *in vivo* system and has not been sub-compartmentalized further. In our model, the lymph node compartment does not receive any blood supply from the plasma, but it acts as a conduit and receives NP mass from the extravascular space of all the healthy compartments (except kidneys) via lymph flow, which are then returned to the plasma compartment.

$V_{\mathrm{LN}}\frac{dC_{\mathrm{LN}}}{dt}=-C_{\mathrm{LN}}\left( L_{B}+L_{H}+L_{\mathrm{LU}}+L_{S}+L_{\mathrm{GI}}+L_{L}+L_{M}+L_{O} \right)+{L_{B}C}_{e,B}+{L_{H}C}_{e,H}+{L_{\mathrm{LU}}C}_{e,LU}+{L_{S}C}_{e,S}+{L_{G}C}_{e,GI}+{L_{L}C}_{e,L}+{L_{M}C}_{e,M}+{L_{O}C}_{e,O}$,

$V_{\mathrm{LN}}C_{\mathrm{LN}}\left( 0 \right)=0$ (S33)

where $C_{\mathrm{LN}}$ represents the concentration of NPs in the lymph node compartment that has a volume $V_{\mathrm{LN}}$ (chosen to be 1 ml).

- 1. **Plasma compartment**

$V_{P}\frac{dC_{P}}{dt}=-C_{P}\left( Q_{B}+Q_{H}+Q_{\mathrm{LU}}+Q_{S}+Q_{\mathrm{GI}}+Q_{\mathrm{HA}}+Q_{K}+Q_{M}+Q_{O}+Q_{T} \right)+\left( Q_{B}-L_{B} \right)C_{v,B}^{f}+\left( Q_{H}-L_{H} \right)C_{v,H}^{f}+\left( Q_{\mathrm{LU}}-L_{\mathrm{LU}} \right)C_{v,LU}^{f}+\left( Q_{L}-L_{L} \right)C_{v,L}^{f}+\left( Q_{K}-\mathrm{GFR} \right)C_{v,K}^{f}+\left( Q_{M}-L_{M} \right)C_{v,M}^{f}+\left( Q_{O}-L_{O} \right)C_{v,O}^{f}+Q_{T}C_{v,T}^{f}+C_{\mathrm{LN}}\left( L_{B}+L_{H}+L_{\mathrm{LU}}+L_{S}+L_{\mathrm{GI}}+L_{L}+L_{M}+L_{O} \right)$,

${V_{P}C}_{P}\left( 0 \right)=100$ (S34)

- 1. **Excreta**

$\frac{dN_{\mathrm{excreta}}}{dt}={k_{\deg}N}_{p,S}, +B{\cdot C}_{e,L}+{k_{\deg}N}_{p,L}+{U\cdot C}_{e,K}+{k_{\deg}N}_{e,T}$,

$N_{\mathrm{excreta}}\left( 0 \right)=0$ (S35)

where $N_{\mathrm{excreta}}$ represents the total excreted mass of NPs.

1. **Equations for hydrodynamic functions** $\boldsymbol{F}$ **and** $\boldsymbol{G}$ [1-4]

$F=1-2.1\alpha_{i}+2\alpha_{i}^{3}-0.9\alpha_{i}^{5}-1.3\alpha_{i}^{6}+3.8\alpha_{i}^{8}-4.1\alpha_{i}^{9}$ (S36)

where $\alpha_{i}$ is the ratio of NP to pore size, i.e. $r/{r_{pore,i}}$.

$G=(1-\frac{2\alpha_{i}^{2}}{3}-0.2\alpha_{i}^{5})/(1-0.7\alpha_{i}^{5})$ (S37)

1. **Approximation of tumor microvascular surface area**

The radius of the spherical tumor $r_{\mathrm{tumor}}$ simulated in our model was chosen to be 5 mm (Table 1), such that the tumor has a volume ≈ 523.6 mm^3^. Given that the reference value of tumor vascular fraction is 0.1 (Table 3), the vascular volume of the tumor is thus ≈ 52.4 mm^3^. The vascular volume represents the volume of blood in the tumor, and assuming that this volume is contained in a hypothetical cylinder representing a single blood capillary of radius $R$ (= 5 μm) and an unknown length x, we can estimate the unknown length x by equating the vascular volume of the tumor (52.4 mm^3^) to the volume of the cylinder ($\pi R^{2}x$), which gives us the value of length x of cylinder ≈ 667178 mm. Now, we can calculate the lateral surface area of the cylinder ($2\pi Rx$) to approximate the total tumor microvascular surface area of the tumor ≈ 20960 mm^2^. For comparison with literature, we calculate the microvascular surface area per unit volume of the tumor to be 20960 mm^2^/523.6 mm^3^ ≈ 40 mm^2^/mm^3^ (Table 2).

1. **Equations for capillary hemodynamics**

To approximate the volumetric blood flow rate in a single capillary (length $l$, radius $R$), we first approximate the total number of capillaries ($n$) in an organ $i$ by dividing the total capillary vascular volume of organ $i$ by the volume of a single capillary ($\pi R^{2}l$), such that:

$n=f_{\mathrm{cap}}V_{v,i}/(\pi R^{2}l)$,

where $f_{\mathrm{cap}}$ is the fraction of vascular volume of an organ that is made up of capillaries (≈ 0.55 [5]).

Next, by dividing the total blood flow rate into organ $i$ by the number of capillaries in organ $i$, we can approximate the blood flow rate through a single capillary as:

$Q_{cap,i}=Q_{i}/(1-H)/(f_{\mathrm{cap}}V_{v,i}/\pi R^{2}l)$ (S38)

Since $Q_{i}$ is the plasma flow rate through organ $i$, the blood rate can be approximated as $Q_{i}/(1-H)$, where $H$ is the hematocrit. Eq. S38 can be rewritten as:

$Q_{cap,i}=\frac{\pi R^{2}lQ_{i}}{(1-H)f_{\mathrm{cap}}V_{v,i}}$ (S39)

The capillary blood flow velocity $u$ can be calculated from Eq. S39 by dividing the capillary blood flow rate $Q_{cap,i}$ by the cross-sectional area of the capillary, such that:

$u=Q_{cap,i}/\pi R^{2}$ (S40)

Further, the mean capillary shear rate $\dot{\gamma}$ can be approximated as [6]:

$\dot{\gamma}=8Q_{cap,i}/(3\pi R^{3})$ (S41)

1. **Equations for cardiac output and organ plasma flow rates**

Equation for calculation of cardiac output for rats is given by [7]:

$CO={0.235\cdot(WT)}^{0.75}$, (S42)

where $\mathrm{CO}$ is the cardiac output in $L\cdot\min^{-1}$ and $\mathrm{WT}$ is the body weight in $\mathrm{kg}$.

From the cardiac output, we can calculate the plasma flow rate into organ $i$ as:

$Q_{i}=f_{CO,i}\cdot CO\cdot(1-H)$ (S43)

where $f_{CO,i}$ is the fractional cardiac output available for organ $i$ (Table 1).

1. **Calculation of organ weights and vascular volumes**

The vascular volume of organ $i$ ($V_{v,i}$) can be calculated as:

$V_{v,i}=f_{v,i}\frac{f_{WT,i}\cdot WT}{\rho_{organ}}$, (S44)

where $f_{WT,i}$ is the fractional body weight of organ $i$, $\rho_{organ}$ is the density of an organ (≈ 1 g·cm^-3^ for all organs), and $f_{v,i}$ is the vascular fraction of the volume of organ $i$.

1. **Calculation of macrophage area fraction**

*Liver*

As per the parameter values given in Table 1, the weight of liver for a reference animal of weight 200 g is ($f_{WT,L}\cdot WT$) = 10 g. Given that the number of macrophages in per gram of liver = 2.72×10^7^ [8], we can estimate the total number of macrophages in liver to be 2.72x10^8^. Further, according to Equation S44 and Table 1, the vascular volume of liver ($V_{v,L}$) is 2.1 ml. Since we know that the capillary volume fraction ($f_{\mathrm{cap}}$) of an organ is 0.55, we can approximate the capillary volume of the liver to be ($f_{\mathrm{cap}}V_{v,L}$) 1.155 ml. Assuming that all the blood in the capillary volume of the liver is contained in a hypothetical capillary of length x and radius 5 μm, we can estimate the microvascular surface area of the liver to be ≈14514 cm^2^ (using the same steps as section 3). Assuming that all the macrophages reside in the microvascular space of the liver, in order to determine the area fraction of microvasculature occupied by macrophages ($A_{mac,L}$), we calculate the ratio of total surface area of macrophages to the microvascular surface area of the liver. Assuming a macrophage to be a sphere of radius ($r_{\mathrm{mac}}$) 15 μm, the surface area of a single macrophage is ≈ 2827 μm^2^, thus $A_{mac,L}=\frac{2827x{10}^{-8}\times2.72x{10}^{8}}{14514} 0.5$.

In the above calculation we assumed that all the macrophages reside in the microvascular space, however a fraction of those macrophages should be in the extravascular space as well. Thus, the above value of $A_{mac,L}$ is an upper bound of macrophage area fraction in the liver microvasculature.

*Spleen*

Given that the macrophages in the spleen are predominantly located in the extravascular space associated with the sinusoids of the red pulp [9], we assume that only 1% of these macrophages have direct access to NPs passing through the blood. Since the number of macrophages in per gram of spleen = 2.08×10^8^ [8], weight of spleen = 2 g, we approximate the total macrophage count in spleen to be ≈ 4.16×10^6^. To estimate the area fraction of splenic microvasculature occupied by macrophages ($A_{mac,S}$), we determined the microvascular surface area of spleen to be ≈ 968 cm^2^ (using the same method as used for liver). Thus, $A_{mac,S}$ that is the ratio of total surface area of macrophages to the microvascular surface area of the spleen ≈ 0.1.

**Supplementary References**

1. Mescam M, Eliat PA, Fauvel C, Certaines JDd, Bézy‐Wendling J. A physiologically based pharmacokinetic model of vascular–extravascular exchanges during liver carcinogenesis: application to MRI contrast agents. Contrast media & molecular imaging. 2007;2(5):215-28.

2. Stylianopoulos T, Soteriou K, Fukumura D, Jain RK. Cationic nanoparticles have superior transvascular flux into solid tumors: insights from a mathematical model. Annals of biomedical engineering. 2013;41(1):68-77.

3. Lightfoot EN, Bassingthwaighte JB, Grabowski EF. Hydrodynamic models for diffusion in microporous membranes. Annals of biomedical engineering. 1976;4(1):78-90.

4. Bungay PM, Brenner H. Pressure drop due to the motion of a sphere near the wall bounding a Poiseuille flow. Journal of Fluid Mechanics. 1973;60(1):81-96.

5. Gould IG, Tsai P, Kleinfeld D, Linninger A. The capillary bed offers the largest hemodynamic resistance to the cortical blood supply. Journal of cerebral blood flow and metabolism : official journal of the International Society of Cerebral Blood Flow and Metabolism. 2017;37(1):52-68. Epub 2016/11/01. doi: 10.1177/0271678x16671146. PubMed PMID: 27780904; PubMed Central PMCID: PMCPMC5363755.

6. Kamoun WS, Chae S-S, Lacorre DA, Tyrrell JA, Mitre M, Gillissen MA, et al. Simultaneous measurement of RBC velocity, flux, hematocrit and shear rate in vascular networks. Nature methods. 2010;7(8):655.

7. Brown RP, Delp MD, Lindstedt SL, Rhomberg LR, Beliles RP. Physiological parameter values for physiologically based pharmacokinetic models. Toxicology and industrial health. 1997;13(4):407-84.

8. Li D, Morishita M, Wagner JG, Fatouraie M, Wooldridge M, Eagle WE, et al. In vivo biodistribution and physiologically based pharmacokinetic modeling of inhaled fresh and aged cerium oxide nanoparticles in rats. Particle and fibre toxicology. 2015;13(1):45.

9. Cataldi M, Vigliotti C, Mosca T, Cammarota M, Capone D. Emerging role of the spleen in the pharmacokinetics of monoclonal antibodies, nanoparticles and exosomes. International journal of molecular sciences. 2017;18(6):1249.
